# Targeting MDM2 for Neuroblastoma Therapy: In Vitro and In Vivo Anticancer Activity and Mechanism of Action

**DOI:** 10.3390/cancers12123651

**Published:** 2020-12-05

**Authors:** Wei Wang, Xinjie Wang, Mehrdad Rajaei, Ji Youn Youn, Atif Zafar, Hemantkumar Deokar, John K. Buolamwini, Jianhua Yang, Jennifer H. Foster, Jia Zhou, Ruiwen Zhang

**Affiliations:** 1 Department of Pharmacological and Pharmaceutical Sciences, College of Pharmacy, University of Houston, Houston, TX 77204, USA; wwang4@central.uh.edu (W.W.); xwang202@central.uh.edu (X.W.); mrajaei@central.uh.edu (M.R.); jyyoun0125@gmail.com (J.Y.Y.); atifzafa@central.uh.edu (A.Z.); 2 Drug Discovery Institute, University of Houston, Houston, TX 77204, USA; 3 Department of Pharmaceutical Sciences, College of Pharmacy, Rosalind Franklin University of Medicine and Science, North Chicago, IL 60064, USA; h.deokar@rosalindfranklin.edu (H.D.); john.buolamwini@rosalindfranklin.edu (J.K.B.); 4 Department of Pediatrics, Texas Children’s Hospital, Baylor College of Medicine, Houston, TX 77030, USA; jianhuay@bcm.edu (J.Y.); jhfoster@txch.org (J.H.F.); 5 Chemical Biology Program, Department of Pharmacology and Toxicology, University of Texas Medical Branch, Galveston, TX 77555, USA; jizhou@utmb.edu

**Keywords:** neuroblastoma, MDM2, p53, SP141, malignancies, metastasis

## Abstract

**Simple Summary:**

Neuroblastoma is a malignant tumor of the sympathetic nervous system that causes aggressive disease in children. The overall survival rate of high-risk patients is very low, therefore developing effective and safe therapies for neuroblastoma is an urgent unmet medical need. The mouse double minute 2 (MDM2) homolog gene is amplified and overexpressed in neuroblastoma and contributes to the poor response to treatment and poor prognosis in patients with high-risk neuroblastoma. Therefore, targeting MDM2 provides a promising approach to neuroblastoma therapy, especially for advanced disease. In the present study, we tested a unique MDM2 inhibitor, SP141, for its therapeutic efficacy and safety in neuroblastoma tumor models. We found that SP141 has significant anti- neuroblastoma activity in cell culture and inhibits tumor growth in animal models of human neuroblastoma, without any noticeable host toxicity. These results provide the basis for targeting MDM2 to treat high-risk neuroblastoma.

**Abstract:**

Background: Neuroblastoma is an aggressive pediatric solid tumor with an overall survival rate of <50% for patients with high-risk disease. The majority (>98%) of pathologically-diagnosed neuroblastomas have wild-type p53 with intact functional activity. However, the mouse double minute 2 (MDM2) homolog, an E3 ubiquitin ligase, is overexpressed in neuroblastoma and leads to inhibition of p53. MDM2 also exerts p53-independent oncogenic functions. Thus, MDM2 seems to be an attractive target for the reactivation of p53 and attenuation of oncogenic activity in neuroblastoma. Methods: In this study, we evaluated the anticancer activities and underlying mechanisms of action of SP141, a first-in-class MDM2 inhibitor, in neuroblastoma cell lines with different p53 backgrounds. The findings were confirmed in mouse xenograft models of neuroblastoma. Results: We demonstrate that SP141 reduces neuroblastoma cell viability, induces apoptosis, arrests cells at the G2/M phase, and prevents cell migration, independent of p53. In addition, in neuroblastoma xenograft models, SP141 inhibited MDM2 expression and suppressed tumor growth without any host toxicity at the effective dose. Conclusions: MDM2 inhibition by SP141 results in the inhibition of neuroblastoma growth and metastasis, regardless of the p53 status of the cells and tumors. These findings provide proof-of-concept that SP141 represents a novel treatment option for both p53 wild-type and p53 null neuroblastoma.

## 1. Introduction

Neuroblastoma is a pediatric extracranial tumor of the sympathetic nervous system and contributes to about 15% of all pediatric cancer deaths [1,2,3,4]. A hallmark of neuroblastoma is high genetic, biological, clinical, and morphological heterogeneity, which can lead to an uneven response to treatment [5]. The International Neuroblastoma Risk Group (INRG) has classified neuroblastoma into very low-risk, low-risk, intermediate-risk, and high-risk groups [6,7]. Treatment for high-risk neuroblastoma is intense and includes multimodal chemotherapy, autologous stem cell transplant, radiation, and immunotherapy [8]. However, the survival rate for children with high-risk neuroblastoma is less than 50%, and nearly half of patients develop drug resistant tumors and suffer relapse [9,10,11,12,13,14]. Thus, it is prudent to identify and develop novel non-toxic treatment strategies for neuroblastoma.

In recent years, research has focused on investigating the genetic and epigenetic changes involved in the tumorigenesis of neuroblastoma. High-throughput genome analyses have identified several genetic aberrations that contribute to neuroblastoma development. Intrinsic alterations at the genetic and epigenetic levels both suggest strategies for molecular targeted therapies, and also impose limitations to the use of such treatment for neuroblastoma [15]. Among the genetic aberrations, amplification of oncogenes and loss of tumor suppressor activity play a significant role in tumorigenesis [16,17]. One of these oncogenes is the human homolog of murine double minute 2 (MDM2, sometimes called HDM2), a negative regulator of p53, which has been found to be amplified in several human malignancies, including neuroblastoma [18,19]. In addition, MDM2 protein overexpression is often present even in neuroblastomas without MDM2 gene amplification, and is linked to a poorer prognosis of patients [18,19]. It has been suggested that the presence of T to G single nucleotide polymorphism (SNP) (SNP309; rs2279744) in the promoter region of MDM2 [20] may increase the MDM2-associated malignant activity and contribute to the development of neuroblastoma [21]. Enhanced MDM2 activity leads to inhibition of the p53 pathway and contributes to tumor formation. Under normal conditions, MDM2 binds to p53 and ubiquitinates it. A study by Slack et al. showed that transcriptional activation of MDM2 via MYCN contributes to the decreased p53 activity in neuroblastoma [22]. However, abnormally post-translated p53 has been found to be resistant to MDM2-mediated degradation in neuroblastoma cells, indicating the presence of impairment of p53 function regardless of high levels in the cells [23]. Therefore, MDM2 targeting rather than p53 would be an effective strategy in neuroblastoma cells [19].

MDM2 has also been found to exhibit non-canonical p53-independent functions that contribute to neuroblastoma growth, progression, and development. In particular, MDM2 stabilizes mRNA of vascular endothelial growth factor (VEGF) by binding directly to 3’ UTR of the mRNA, thus in turn causes the increased translation of VEGF, contributing to the growth of neuroblastoma under hypoxia condition [24]. The ring domain of MDM2 binds to the MYCN mRNA adenylate/uridylate-rich elements (AREs) within the 3’UTR, and thereby increases the MYCN mRNA stability and translation in neuroblastoma cells [25]. In addition, elevated MDM2 expression has also been found to promote multidrug resistance in neuroblastoma cells [26]. Overall, these studies suggest MDM2 is a potential target for anticancer therapy in neuroblastoma [19].

Since the majority of neuroblastomas harbor high levels of MDM2, it is critical to develop MDM2 inhibitors for neuroblastoma treatment. The ideal MDM2 inhibitor should exert anticancer activity in neuroblastoma cells, independent of their p53 status (wild-type, null, or mutated). Other research groups have identified nutlin-3 [27], MI-77301 [28], MI-63 [29], RITA [30], and RG7112 [31] as MDM2 inhibitors that exhibit anticancer activity in neuroblastoma cells. In addition, a study by Giustiniano et al. has identified ‘compound 12’ as a dual inhibitor of MDM2/p53 and MDM4/p53 complexes, which increases p53 gene expression and induces apoptosis in SH-SY5Y neuroblastoma cells [32]. However, none of these previously-identified agents has yet been accepted as a clinical treatment for neuroblastoma. Keeping in view the above facts, the aim of the present study was to evaluate the anti-neuroblastoma activity of SP141 (formal chemical name: 6-methoxy-1-(1-naphthalenyl)-9*H*-pyrido(3,4-*b*)indole) [33,34,35,36], a first-in-class MDM2 inhibitor with unique mechanisms of action different from the existing MDM2 inhibitors. This study provides initial support for using SP141 as a therapeutic agent for neuroblastoma, irrespective of the p53 background of the individual tumors.

## 2. Results

### 2.1. SP141 Inhibits MDM2 in Neuroblastoma Cells, Independent of p53

**Figure 1:**
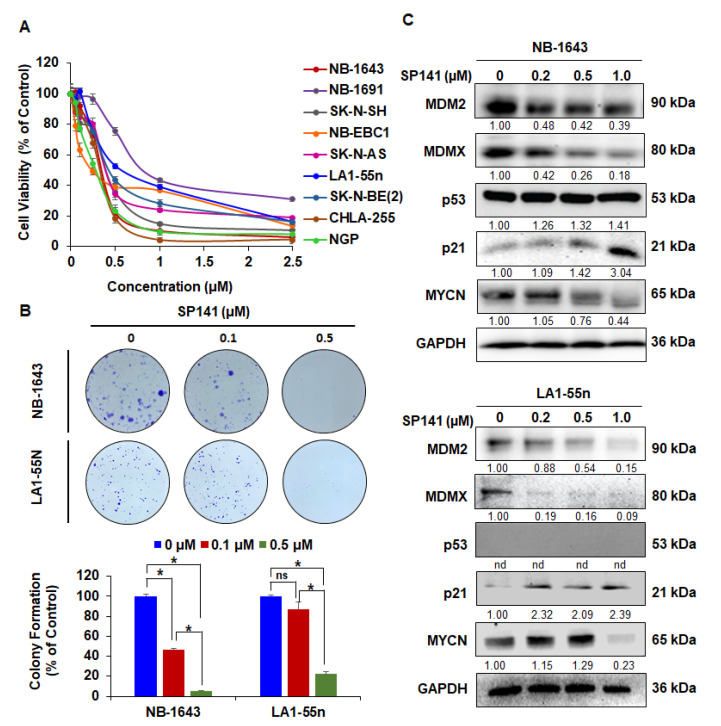
SP141 inhibits cell growth and reduces MDM2 protein levels in neuroblastoma cells, independent of p53. (**A**) SP141 inhibits the growth of neuroblastoma cells, independent of p53. The NB-1643 (p53 wild-type), SK-N-SH (p53 wild-type), NB-EBC1 (p53 wild-type), CHLA255 (p53 wild-type), NGP (p53 wild-type), SK-N-AS (p53 mutation), LA1-55n (p53 null), and two multidrug-resistant neuroblastoma cell lines NB-1691 (p53 wild-type) and SK-N-BE2 (p53 mutation) were treated with various concentrations of SP141 (0–2.5 µM) for 72 h. The cell viability was analyzed by the MTT assay, and the 50% inhibitory concentration values (IC_50_) were calculated. (**B**) SP141 inhibits the colony formation of neuroblastoma cells, independent of p53. The NB-1643 and LA1-55n cells were treated with various concentrations of SP141 (0, 0.1, and 0.5 µM) for 24 h. Ten days after drug removal, cells were fixed and stained with crystal violet, and images were prepared. (**C**) SP141 reduces the MDM2 protein levels in neuroblastoma cells, independent of p53. The NB-1643 and LA1-55n cells were treated with various concentrations of SP141 (0, 0.25, 0.5, and 1 µM) for 24 h. The expression levels of MDM2 and related proteins were detected by Western blotting. All band intensities were quantitated using the ImageJ software, and the results were normalized to the control lane for each target. “nd” denotes “not detected”. All assays were performed in triplicate, and all the experiments were repeated at least three times. Representative data are shown. When applicable, the data were analyzed by two-sided Student’s *t*-test and are shown as the means ± SEM. (* *p* < 0.01, and “ns” denotes “not significant”).

SP141 was investigated for inhibitory effects on cell viability in a panel of neuroblastoma cells with different genetic backgrounds of p53: NB-1643 (p53 wild-type (WT)), SK-N-SH (p53 WT), NB-EBC1 (p53 WT), CHLA-255 (p53 WT), NGP (p53 WT), SK-N-AS (p53 mutation (MT)), LA1-55n (p53 null), and two multidrug-resistant neuroblastoma cells NB-1691 (p53 WT) [37] and SK-N-BE(2) (p53 MT) [38] (Table 1). The effects of SP141 on the viability of these cells were evaluated using the 3-(4,5-dimethylthiazol-2-yl)-2,5-diphenyl tetrazolium bromide (MTT) assay. As shown in Figure 1A and Table 1, SP141 significantly reduced neuroblastoma cell viability, with IC_50_ values ranging from 0.26 to 0.89 µM, regardless of the p53 status of the cells. As we reported earlier, SP-141 had much less activity against the normal lung fibroblast cell line IMR90 (IC_50_: 13.22 µM) [34], with the IC_50_ value being >14.9–58.4 times higher than that of cancer cells, indicating its specificity against neuroblastoma cells. Then, we used NB-1643 and LA1-55n cell lines to further explore the underlying molecular mechanisms responsible for SP141’s anti-neuroblastoma activity. We demonstrated that SP141 inhibited cancer cell colony formation in a concentration-dependent manner in both cell lines (Figure 1B). The effects of SP141 on MDM2 and related proteins expression were analyzed. As shown in Figure 1C, the MDM2 protein levels were decreased in a concentration-dependent manner in both the NB-1643 and LA1-55n cell lines. It is important to note that treatment with SP141 also decreased MDMX in both cell lines, with a more pronounced decrease in LA1-55n neuroblastoma cells (Figure 1C). As the results of the MDM2 inhibition, SP141 also increased the expression level of wild-type p53 in NB-1643 cells and of p21 in both cell lines. Since MDM2 plays a p53-independent role in the regulation of MYCN mRNA stabilization and translation [25], the MYCN levels were also evaluated in neuroblastoma cells with and without SP141 treatment. As shown in Figure 1C, in both neuroblastoma cell lines, SP141 significantly inhibited MYCN expression at the 1.0 µM concentration.

### 2.2. SP141 Induces Apoptosis and Cell Cycle Arrest in Neuroblastoma Cells

**Figure 2:**
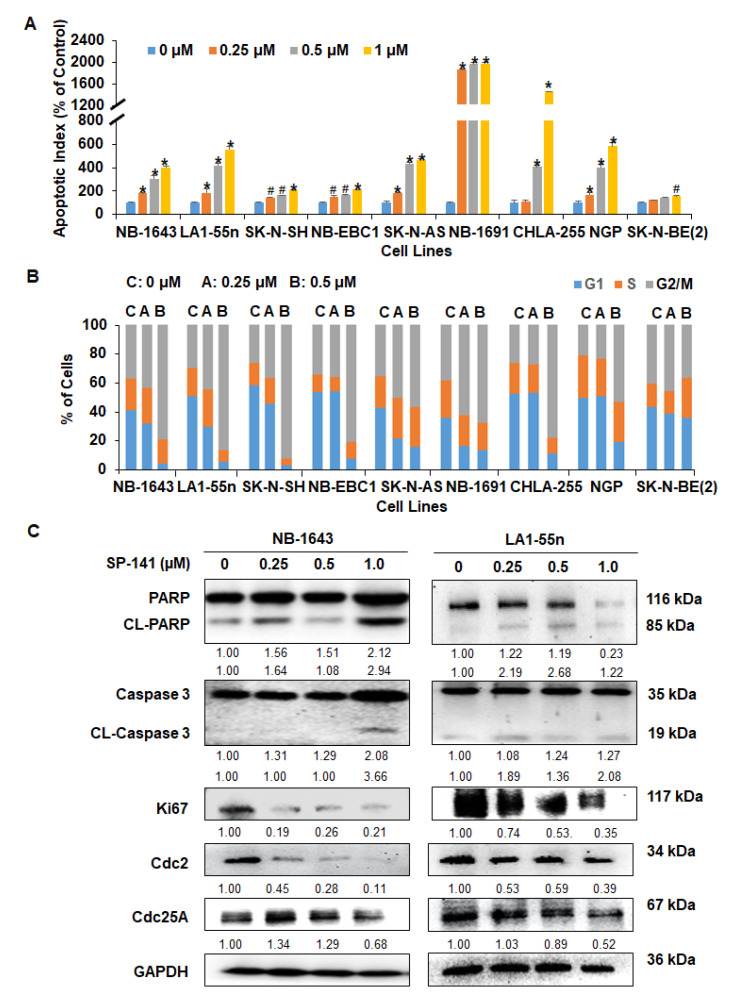
SP141 induces apoptosis and cell cycle arrest in neuroblastoma cells, independent of p53. (**A**) SP141 induces apoptosis in neuroblastoma cells. (**A**) The NB-1643 (p53 wild-type), SK-N-SH (p53 wild-type), NB-EBC1 (p53 wild-type), CHLA255 (p53 wild-type), NGP (p53 wild-type), SK-N-AS (p53 mutation), LA1-55n (p53 null), and two multidrug-resistant neuroblastoma cell lines NB-1691 (p53 wild-type) and SK-N-BE2 (p53 mutation) were treated with various concentrations of SP141 (0, 0.25, 0.5, and 1 µM) for 48 h. The cell apoptosis was measured by the Annexin V-FITC method. (**B**) Neuroblastoma cells were treated with various concentrations of SP141 (0, 0.25, and 0.5 µM) for 24 h. The cell cycle distribution was assessed by PI staining. (**C**) NB-1643 and LA1-55n cells were treated with various concentrations of SP141 (0, 0.25 0.5, and 1 µM) for 24 h. The expression of proteins related to apoptosis and cell cycle arrest was examined by Western blotting. All band intensities were quantitated using the ImageJ software, and data were normalized to the control lane for each target. All assays were performed in triplicate, and all experiments were repeated at least three times. Representative data are shown. When applicable, the data were analyzed by two-sided Student’s *t*-test and are shown as the means ± SEM. (^#^ *p* < 0.05, * *p* < 0.01).

SP141 was further evaluated for its effects on apoptosis and cell cycle progression in all neuroblastoma cell lines. As shown in Figure 2A, SP141 treatment significantly increased apoptosis in all neuroblastoma cell lines, independent of p53 status. At 1 µM, SP141 increased the cell apoptotic index from 1.56-fold (*p* < 0.01) to 19.77-fold (*p* < 0.01), compared to the levels in control cells. In addition, with 0.5 µM being the most effective SP141 concentration examined, SP141 induced cell cycle arrest at the G2/M phase in all cell lines, except for the SK-N-BE (2) cells (Figure 2B). In the SK-N-BE (2) cells, SP141 induced cell cycle arrest at the G2/M phase at the lower concentration but displayed an S phase arrest at the higher concentration (Figure 2B). We further examined the expression of apoptosis-related proteins following SP141 treatment in NB-1643 and LA1-55n cell lines. As shown in Figure 2C, SP141 treatment increased the expression of cleaved Caspase 3 and cleaved PARP in both cell lines. In addition, consistent with the cell cycle results, SP141 treatment led to decreased expression of Cdc2 and Cdc25A in both NB-1643 and LA1-55n neuroblastoma cells. We also determined the protein level of Ki67, a cell proliferation marker, and observed that SP141 treatment decreased the expression level of Ki67 in a p53-independent manner (Figure 2C).

### 2.3. SP141 Inhibits the Migration of Neuroblastoma Cells

**Figure 3:**
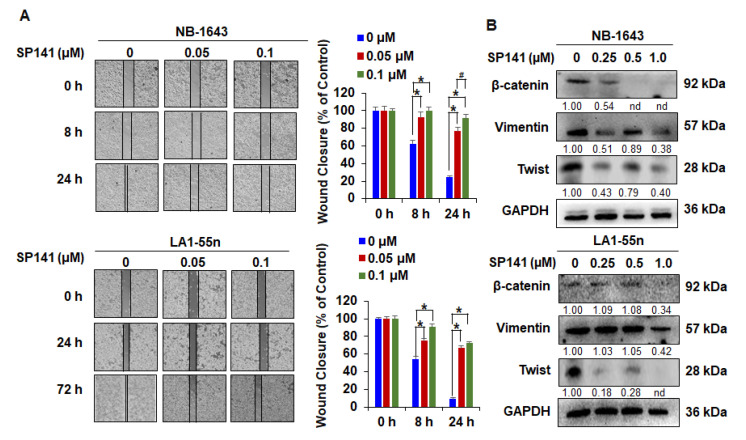
SP141 inhibits the migration of neuroblastoma cells. (**A**) NB-1643 and LA1-55n cells were grown to confluence in a six-well plate and a scratch was made at experimental time zero. The cells were exposed to various concentrations of SP141, and the wells were imaged at different time points. Graphs (right panel) show the quantitative results of wound closure. (**B**) NB-1643 and LA1-55n cells were treated with various concentrations of SP141 for 24 h, then Western blot analyses were performed to assess the expression of epithelial-mesenchymal transition (EMT)-related molecular markers. All band intensities were determined using the ImageJ software and are shown normalized to the control lane for each target. “nd” denotes “not detected”. All assays were performed in triplicate, and all experiments were repeated at least three times. Representative data are shown. Where applicable, the data were analyzed by Student’s *t*-test and results are shown as the means ± SEM. (^#^ *p* < 0.05, * *p* < 0.01).

Cancer cell migration is an important property of tumor cell invasion and promotes the metastatic potential of malignant cells [39]. A wound healing assay was performed to examine the effects of SP141 on the migration of NB-1643 and LA1-55n neuroblastoma cells. In this assay, cells were treated with a low concentration (0.05 or 0.1 µM) of SP141 to avoid effects on cell proliferation. The wound made in the monolayer was evaluated at different time points. As shown in Figure 3A, control NB-1643 cells closed the wound at around 24 h, whereas control LA1-55n cells closed the wound at around 72 h. As shown in Figure 3A, SP141 treatment significantly decreased the migration of both neuroblastoma cell lines in a concentration-dependent manner, with the 0.1 µM concentration more effectively preventing migration (* *p* < 0.01). The cancer cell migration in the absence or presence of SP141 treatment was also analyzed by investigating epithelial-mesenchymal transition (EMT)-related molecular markers. As evident from these Western blotting experiments (Figure 3B), SP141 treatment resulted in concentration-dependent decreases in the expression of β-catenin, Vimentin, and Twist.

### 2.4. SP141 Inhibits Neuroblastoma Xenograft Tumor Growth 

**Figure 4:**
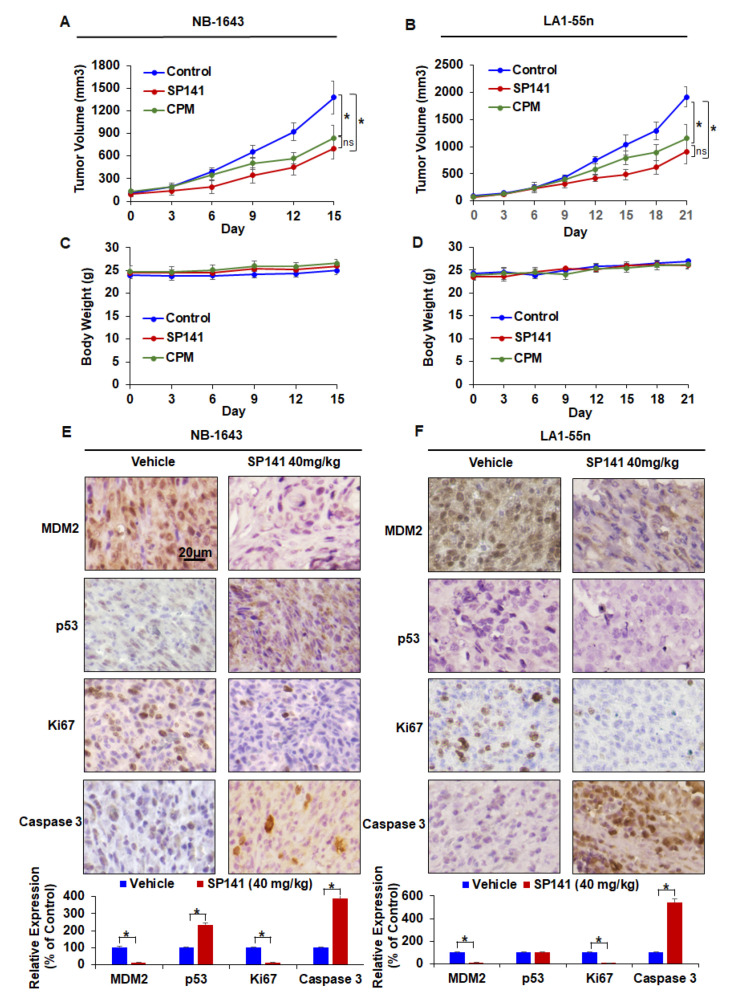
SP141 inhibits neuroblastoma xenograft tumor growth in vivo. Nude mice bearing NB-1643 and LA1-55n xenograft tumors were treated with SP141, which was administered by i.p. injection at 40 mg/kg/day, 5 d/week for 15 and 21 days, respectively. (**A**,**B**) Tumor growth curves of NB-1643 and LA1-55n xenograft tumors are shown. Each point on the line represents the mean tumor volume, and the bars represent the SEM. (**C**,**D**) Animals were monitored for changes in body weight as a surrogate marker for toxicity in both xenograft models. Each point on the line represents the mean body weight and the bars represent the SEM. (**E**,**F**) At the end of the experiments, NB-1643 and LA1-55n xenograft tumors were removed and further analyzed for their protein expression by immunohistochemistry (scale bar, 20 μm). Graphs (lower panel) show the quantification of the positive cells. The data were analyzed by an ANOVA with post-hoc test and are shown as the means ± SEM. (* *p* < 0.01, and “ns” denotes “not significant).

To determine if SP141 could inhibit tumor growth under in vivo conditions, we next examined its efficacy in NB-1643 and LA1-55n xenograft models. As shown in Figure 4A, nude mice bearing NB-1643 xenograft tumors were treated with cyclophosphamide (CPM; as a positive control) or SP141 (40 mg/kg/day, 7 days/week) by intraperitoneal (i.p.) injection for 15 days, resulting in 39.1% and 49.2% inhibition of tumor growth, respectively, compared to vehicle-treated mice. To further demonstrate the in vivo efficacy of SP141, nude mice bearing LA1-55n xenograft tumors were treated with CPM and SP141 (40 mg/kg/day, 7 days/week) by intraperitoneal injection for 21 days. As shown in Figure 4B, CPM and SP141 inhibited the LA1-55n xenograft tumor growth by 38.7% and 52.4%, respectively. Although there was a trend to show SP141 was more effective based on the data presented in the figure, there were no statistically significant differences between the SP141 and CPM treatment groups in either the NB-1643 or LA1-55n xenograft model (Figure 4A,B). Of note, SP141 treatment did not lead to any significant loss of body weight in either model, suggesting that the compound is safe at a dose of up to 40 mg/kg 5 days/week for at least three weeks (Figure 4C,D).

We then examined whether SP141’s inhibitory effects on MDM2 also occurred in vivo. The mechanism underlying the anticancer activity of SP141 in vivo was evaluated by immunohistochemical staining. The protein levels of MDM2, p53, and proteins related to apoptosis and cell cycle progression in the NB-1643 and LA1-55n xenograft models were examined after SP141 treatment. As shown in Figure 4E,F, IHC showed that the MDM2 levels were dramatically decreased in the tumors of SP141-treated mice compared with the controls. Treatment with SP141 at 40 mg/kg/day also significantly reduced the expression levels of Ki67 and increased the expression level of Caspase 3 in both NB-1643 and LA1-55n tumor tissues. In addition, SP141 treatment increased the p53 protein levels in NB-1643 tumors. These results corroborated with the in vitro Western blotting results in both cell lines. Overall, these observations demonstrate that SP141 inhibits MDM2 expression and suppresses neuroblastoma tumor growth in vivo, independent of the p53 status of the tumor.

**Figure 5:**
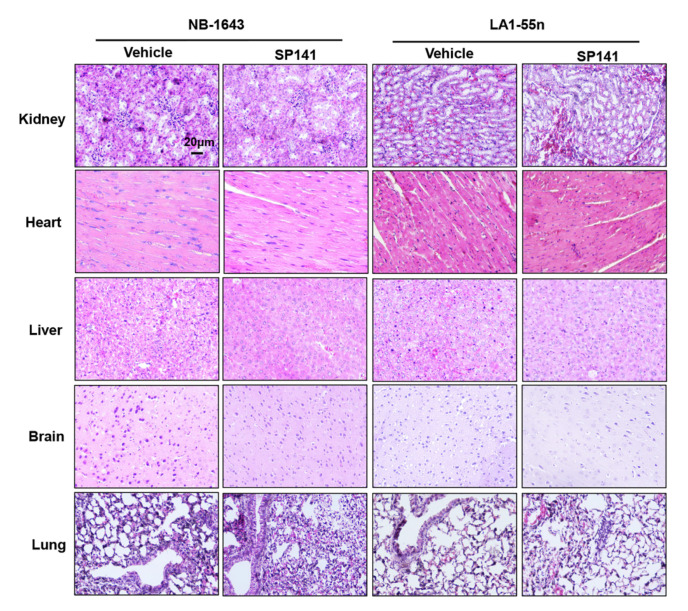
SP141 inhibits neuroblastoma xenograft tumor growth in vivo, without observable toxicity in the animals. Nude mice bearing NB-1643 or LA1-55n xenograft tumors were treated with SP141, which was administered by i.p. injection at 40 mg/kg/day, 5 d/week for 15 and 21 days, respectively. At the termination of the experiments, H&E staining was performed on paraffin-embedded sections of major organs (kidneys, heart, liver, brain, and lungs) from mice bearing NB-1643 and LA1-55n xenograft tumors (scale bar, 20 μm).

Although the lack of weight loss in the mice suggested that SP141 was safe at the dose administered, additional in-depth pathological studies were conducted to determine whether there was any toxicity to specific organ system. The histopathological examination indicated that SP141 treatment did not result in any significant organ abnormalities in either tumor models (Figure 5).

## 3. Discussion

MDM2 is amplified in a variety of malignancies, including neuroblastoma [18,19,28]. The overexpression of MDM2 is linked to a poor prognosis for patients with cancer [18,19,28]. Enhanced MDM2 expression leads to inhibition of the p53 pathway and tumor growth acceleration. In addition, MDM2 has been found to exhibit p53-independent roles in the growth and progression of neuroblastoma. Our lab has a long history of developing novel strategies to target MDM2 for cancer therapy and prevention [40,41,42]. In the past, we have identified natural product MDM2 inhibitors such as genistein [43], curcumin [44], and ginsenosides [45,46,47,48,49,50,51,52], and also discovered small-molecule synthetic MDM2 inhibitors such as the SP series [33,34,35,36,53,54,55,56] and synthetic iminoquinones [57,58,59,60,61,62], which have proven effective against several different malignancies. The present study is the first to report the in vitro and in vivo anti-neuroblastoma effects of SP141, a potent and selective MDM2 inhibitor discovered in our lab. Our previous studies demonstrated that SP141 inhibits cell growth, induces apoptosis and cell cycle arrest, inhibits cell migration and invasion, and induces tumor regression without observable toxicity in models of breast cancer [33], pancreatic cancer [34], hepatocellular carcinoma [35], and glioblastoma [36]. It is most likely SP141 is a target-specific anticancer agent that may have a broad-spectrum of activity against MDM2-overexpressing cancers/tumors. Mechanistically, SP141 inhibits MDM2’s oncogenic functions via both p53-dependent and -independent mechanisms. These effects are believed to be due to the fact that SP141 directly binds to MDM2 with high affinity and induces its autoubiquitination and proteasomal degradation [33,34,35,36].

The present study showed that SP141 significantly reduced neuroblastoma cell viability, inhibited cancer colony formation, induced apoptosis, and arrested the cancer cells in the G2/M phase, and all these effects were independent of p53 status. SP141 effectively downregulated MDM2 expression, as well as MDMX expression, in neuroblastoma tumor cells, regardless of the p53 status of the cells. SP141 treatment also increased p21 expression in neuroblastoma cells, irrespective of their p53 status. This was consistent with the findings of previous reports showing that MDM2 interacts with p21 and acts as a negative regulator of p21 by reducing its protein stability, independent of p53 [63,64]. It has been demonstrated that MDM2 plays a p53-independent role in the regulation of MYCN mRNA stabilization and translation in neuroblastoma cells [25]. Our results showed that SP141 treatment decreased the MYCN expression in both p53 wild-type and p53 null neuroblastoma cells, and this may explain how SP141 inhibits the MDM2 expression in neuroblastoma cells. Our in vitro studies also showed that SP141 exhibits anti-metastatic effects, as evidenced by the results of the wound healing assay and decreases in the expression of EMT-related proteins such as β-catenin, vimentin, and Twist.

In addition, SP141 effectively inhibited the growth of neuroblastoma xenograft tumors in vivo and inhibited MDM2 expression in the tumor tissues and increased the Caspase 3 expression in both NB-1643 and LA1-55n xenograft models, independent of the p53 status. It is also important to note that SP141 exhibited no significant toxicity in mice at the relatively high dose of 40 mg/kg, as indicated by the results of organ specific histopathological examination and tracking of body weight. Overall, our results clearly suggest that SP141 exerts antitumor activity in models of neuroblastoma, and the antitumor activity may mechanistically be due to its targeting MDM2 and inhibiting MDM2 expression, which occurs regardless of the p53 status of the cancer cells.

Previous research has identified several dual inhibitors of the MDM2/p53 complex and MDM4/p53 complex in cancer cells. For instance, RITA inhibited growth, induced apoptosis, and disrupted the interaction between p53 and MDM2/MDMX in neuroblastoma cells, and also inhibited the growth of SK-N-DZ xenograft tumors in mice [30]. Compound 12 is another molecule found to be a dual inhibitor of MDM2/p53 and MDM4/p53 complexes, which also increases p53 protein levels and enhances the levels of p53 target genes (MDM2, p21, PUMA), and inhibits the proliferation of SHSY-5Y neuroblastoma cells [32]. Likewise, SP141 has also been found to reduce the protein levels of both MDM2 and MDMX (MDM4) in neuroblastoma cells. Thus, SP141 appears to act as a dual inhibitor of both MDM2 and MDMX in neuroblastoma cells, irrespective of their p53 status.

This study suggests that SP141 warrants further investigation as an MDM2 antagonist, particularly in combination with other agents currently used to treat neuroblastoma, such as mTOR or ALK inhibitors, to provide improved anticancer activity against neuroblastomas with different genetic backgrounds. In addition, although the anti-neuroblastoma activities of SP141 have been demonstrated in this study, further studies are necessary to confirm the efficacy and safety of SP141 using other models of neuroblastoma, including primary tumor-derived models with different genetic backgrounds.

## 4. Materials and Methods

### 4.1. Chemicals, Reagents, and Cell Lines

SP141 (6-methoxy-1-(1-naphthalenyl)-9*H*-pyrido(3,4-*b*)indole) was synthesized as described previously [33,34,35,36], and the structure of the compound was confirmed by melting point, UV, IR, MS, ^1^H NMR, and ^13^C NMR spectroscopy. The purity of the synthesized compound was estimated to be greater than 99%. All other chemicals and solvents were of the highest grade available and procured from Sigma-Aldrich Company.

Three of the human neuroblastoma cell lines (LA1-55n, SK-N-SH, and SK-N-AS) were purchased from the American Type Culture Collection (ATCC). The NB-1643 and NB-EBC1 cell lines were obtained from Texas Tech University Health Science Center, Children’s Oncology Group. NB-1691 cells were graciously provided by Dr. Andrew Davidoff’s laboratory at St. Jude’s Hospital for Children. SK-N-BE (2), CHLA-255, and NGP cell lines were kindly provided by Dr. Jianhua Yang from Baylor College of Medicine, Texas Children’s Hospital. All cell culture media contained 10% FBS and 1% penicillin–streptomycin unless otherwise specified. NB-1643 and LA1-55n cells were maintained in RPMI-1640 medium; NB-1691 cells were cultured in RPMI-1640 medium containing 2 mM of glutamine; SK-N-AS cells were maintained in DMEM containing 0.1 mM Non-Essential Amino Acids (NEAA); SK-N-SH and SK-N-BE2 cells were cultured in EMEM; NB-EBC1 cells were cultured in IMDM supplemented with 20% fetal bovine serum (FBS), 4mM L-Glutamine, and 1X ITS (5 µg/mL insulin, 5 µg/mL transferrin, and 5 ng/mL selenous acid). CHLA-255 cells were cultured in IMDM medium, and NGP cells were maintained in DMEM medium. Cell culture media and phosphate-buffered saline (PBS) were obtained from Hyclone (Logan, UT). FBS (Gibco) and other supplements were obtained from Thermo Fisher Scientific (Waltham, MA, USA). Penicillin/streptomycin was purchased from Corning (Manassas, VA, USA). The anti-human MDM2 (Ab-2) and p21 (Ab-1) antibodies were from Calbiochem (Billerica, MA, USA). The anti-human PARP1 (H-250), β-catenin (12F7), Twist (Twist2C1a), Cdc2 (17), and Cdc25A (F-6) antibodies were from Santa Cruz Biotechnology Inc. (Santa Cruz, CA, USA). The anti-human p53 (Ab-6) antibody was from EMD Chemicals Inc. (Gibbstown, NJ, USA). The anti-human Ki67 (SP6) antibody was from Abcam (Cambridge, MA, USA). The anti-human MDMX (8C6) Vimentin (V9) antibody was from Sigma-Aldrich (St Louis, MO, USA). The Caspase 3 (9662) antibody was from Cell Signaling (Danvers, MA, USA). The anti-human MYCN (NMYC-1) and GAPDH antibodies were from Novus Biologicals (Littleton, CO, USA).

### 4.2. Assays for the In Vitro Effects of SP141

The cytotoxicity of SP141 on human neuroblastoma cells was evaluated using the MTT reduction assay, as described previously [33,34,35,36]. Briefly, cancer cells were seeded in 96-well plates at a density 1 × 10^3^ cells per well. Cells were treated with various concentrations of SP141 (0, 0.01, 0.1, 0.25, 0.5, 1.0, and 2.5 µM) for 72 h at 37 °C. After incubation with SP141, 10 µL of MTT dye (5 mg/mL) were added to each well, and the plates were incubated for 3–4 h at 37 °C. After this, the supernatant was removed, and the formazan crystals were dissolved in 100 µL of DMSO. Absorbance was recorded at 570 nm using a SpectraMax iD5 Multi-mode microplate reader (Molecular Devices, San Jose, CA, USA).

The cytotoxic effects of SP141 were also evaluated via a colony formation assay [33,34,35,36]. Briefly, 800 cells were seeded in a 6-well culture plate and later treated with various concentrations of SP141 (0, 0.1, and 0.5 µM). After a 24 h treatment, the media was removed and replaced with new culture media, and the cells were incubated for another 10 days. Later, the cells were fixed with fixative (7 parts methanol: 1 part glacial acetic acid), and then stained with crystal violet (0.2 g/L).

Apoptosis was detected using an Annexin V-FITC apoptosis detection [33,34,35,36]. Briefly, cells were seeded at a density of 1 × 10^5^ cells per well in a 6-well plate and treated with various concentrations of SP141 (0, 0.25, 0.5, and 1.0 µM) for 48 h. After treatment, the cells were trypsinized, washed with PBS, and resuspended in 500 µL Annexin V binding buffer. This was followed by adding 5 µL of Annexin and 5 µL of propidium iodide. The samples were analyzed on a BD Accuri C6 Flow cytometer, and the results are plotted to determine the apoptotic index.

The effects of SP141 treatment on the cell cycle distribution were evaluated using propidium iodide (PI) staining [33,34,35,36]. Briefly, 1 × 10^5^ cells per well were seeded in a 6-well plate, and cells were treated with various concentrations of SP141 (0, 0.25, and 0.5 µM) for 24 h. Subsequently, the cells were trypsinized, washed with PBS, and fixed in 95% ethanol at 4 °C overnight. Later, the fixed cells were incubated with RNase and stained with propidium iodide (40 µg/mL), and the DNA contents were analyzed via flow cytometry.

The migration of neuroblastoma cells was measured via the wound healing assay [33,35,36]. Neuroblastoma cells were grown as a monolayer in 6-well culture plates to confluence. After the cells reached confluence, a scratch was made in each well using a pipette tip, and then cells were exposed to various concentrations of SP141 (0, 0.05, and 0.1 µM) with serum-free medium. In order to monitor the cell movement into the wounded area, five fields of each of the three wounds analyzed per condition were photographed at different time points. The cells that migrated to the wounded area were counted using Image-Pro Plus 7.0 software (Media Cybernetics, Rockville, MD, USA).

### 4.3. Western Blotting

NB-1643 and LA1-55n cells were exposed to various concentrations of SP141 (0, 0.25, 0.5, 1.0 µM) for 24 h. After treatment, the cells were trypsinized, washed with PBS, and lysed in RIPA buffer. After centrifugation, equal amounts of protein were resolved by SDS-PAGE and then transferred to trans-Blot nitrocellulose membranes (Bio-Rad Laboratories, Hercules, CA, USA), as described previously [33,34,35,36]. The blots were blocked in 5% non-fat dry milk dissolved in Tris-buffered saline, and the membranes were incubated with primary antibody at 4 °C overnight with gentle shaking, then the blots were exposed to secondary antibodies. The proteins of interest were identified using a digital gel imaging system (UVP ChemStudio Touch, Analytik Jena US, Upland, CA, USA). All band intensities were quantitated using the ImageJ software. The full western blots could see Figure S1.

### 4.4. In Vivo Xenograft Model for Human Cancer

The animal protocol was approved by the Institutional Animal Use and Care Committee of the University of Houston, conforming to the US National Institutes of Health Guide for the Care and Use of Laboratory Animals (PROTO202000081). Male and female athymic pathogen-free nude mice (nu/nu, 4–6 weeks) were purchased from Envigo. To establish NB-1643 and LA1-55n human neuroblastoma xenografts, 5 × 10^6^ cells (total volume of 0.1 mL) were injected subcutaneously into the left inguinal area of the mice. All animals were monitored for activity, physical condition, body weight, and tumor growth. The tumor size was determined by caliper measurement of two perpendicular diameters of the implant every 3 days. The tumor volume (mm^3^) was calculated using the formula: 1/2 × a × b^2^, where ‘a’ is the long diameter and ‘b’ is the short diameter (in cm). The animals bearing human cancer xenografts were randomly divided into treatment and control groups (10 mice per group). The untreated control group received the vehicle only. SP141 was dissolved in PEG400:ethanol:saline (57.1:14.3:28.6, *v/v/v*) and was administered by i.p. injection at a dose of 40 mg/kg/day, 7days per week [33,34,35] for 15 days (NB-1643 tumors) or 21 days (LA1-55n tumors). At the end of the experiments, xenograft tumors and other organs were removed, weighed, and snap-frozen for immunohistochemistry and hematoxylin and eosin (H&E) staining.

### 4.5. H&E Staining and Immunohistochemistry

The tissue specimens were fixed, paraffin-embedded, and cut into 3–5 µm sections. The sections were then deparaffinized and stained with hematoxylin and eosin solution [33,34,35,36]. After staining, the sections were dehydrated and mounted with Permount, and then visualized under a phase contrast microscope. For immunohistochemical staining, the tumor sections were fixed, embedded in paraffin, cut into sections, and affixed onto slides. The tissue slides were incubated with primary antibodies against MDM2, p53, Ki67, or Caspase 3, and then were stained with DAB chromogen as per the instructions included with the DACO Animal Research kit. The sections were counterstained with hematoxylin and were analyzed under a phase-contrast microscope [33,34,35,36]. The positive cells were counted in five different visual areas, and the total area of positive staining was measured using Image-Pro Plus 7.0 (Media Cybernetics, Rockville, MD, USA).

### 4.6. Statistical Analysis

All experimental values were reported as the means ± SEM of three independent experiments. Data were analyzed using GraphPad software V6 (GraphPad Software, San Diego, CA, USA). Student’s *t*-test was used to compare differences between two groups, and an ANOVA with post-hoc test was used to compare differences among three groups. The differences with values of *p* < 0.05 were considered statistically significant.

## 5. Conclusions

In summary, SP141 is a potent MDM2 inhibitor that exhibits anticancer activity against neuroblastoma cells and xenograft tumors, irrespective of their p53 status. We expect that this study will generate a novel candidate for neuroblastoma therapy, which would have a major impact on the treatment of patients with high-risk neuroblastoma.

## Figures and Tables

**Table 1:** IC_50_ of SP141 in neuroblastoma cell lines of varying p53 status.

**Neuroblastoma Cell Lines**			
**Cell Lines**	**p53 Status**	**Multidrug-Resistant**	**IC_50_ (μM)**
NB-1643	WT	−	0.36
SK-N-SH	WT	−	0.32
NB-EBC1	WT	−	0.26
CHLA-255	WT	−	0.42
NGP	WT	−	0.30
NB-1691	WT	Yes [37]	0.89
LA1-55n	Null	−	0.62
SK-N-AS	MT	−	0.41
SK-N-BE (2)	MT	Yes [38]	0.29
**Normal Fibroblast Cell Line**			
**Cell Line**	**p53 Status**	**Drug-Resistant**	**IC_50_ (μM)**
IMR90	WT	−	13.22 [34]

Abbreviation: WT, wild type; MT, mutation.

## Supplementary Materials

The following are available online at https://www.mdpi.com/2072-6694/12/12/3651/s1, Figure S1: The full western blots.
