# Supplementary material for: Targeting MDM2 for Neuroblastoma Therapy: In Vitro and In Vivo Anticancer Activity and Mechanism of Action
Source: Cancers (Basel). 2020 Dec 5;12(12):3651. doi: 10.3390/cancers12123651 (PMC7762001; doi:10.3390/cancers12123651)
Supplement: Supplementary file 1 [file cancers-12-03651-s001.pdf]

Supplementary Materials: Targeting MDM2 for Neuroblastoma Therapy: In Vitro and In Vivo Anticancer Activity and Mechanism of Action

Wei Wang, Xinjie Wang, Mehrdad Rajaei, Ji Youn Youn, Atif Zafar, Hemantkumar Deokar, John K. Buolamwini, Jianhua Yang, Jennifer H. Foster, Jia Zhou and Ruiwen Zhang

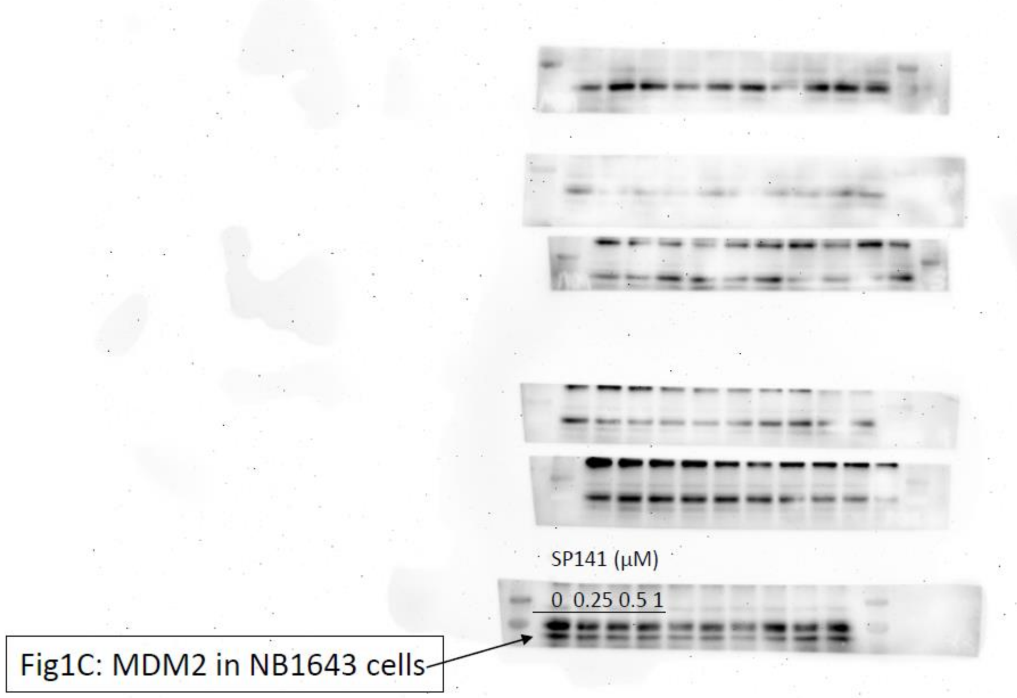

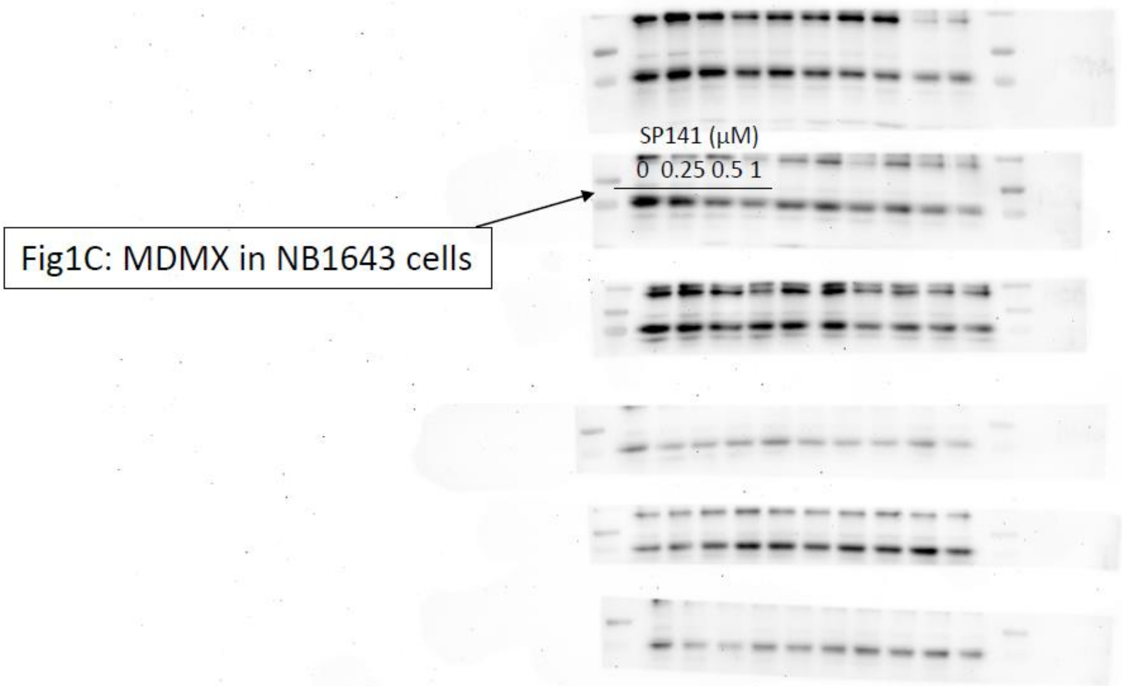

Fig1C: MDMX in NB1643 cells

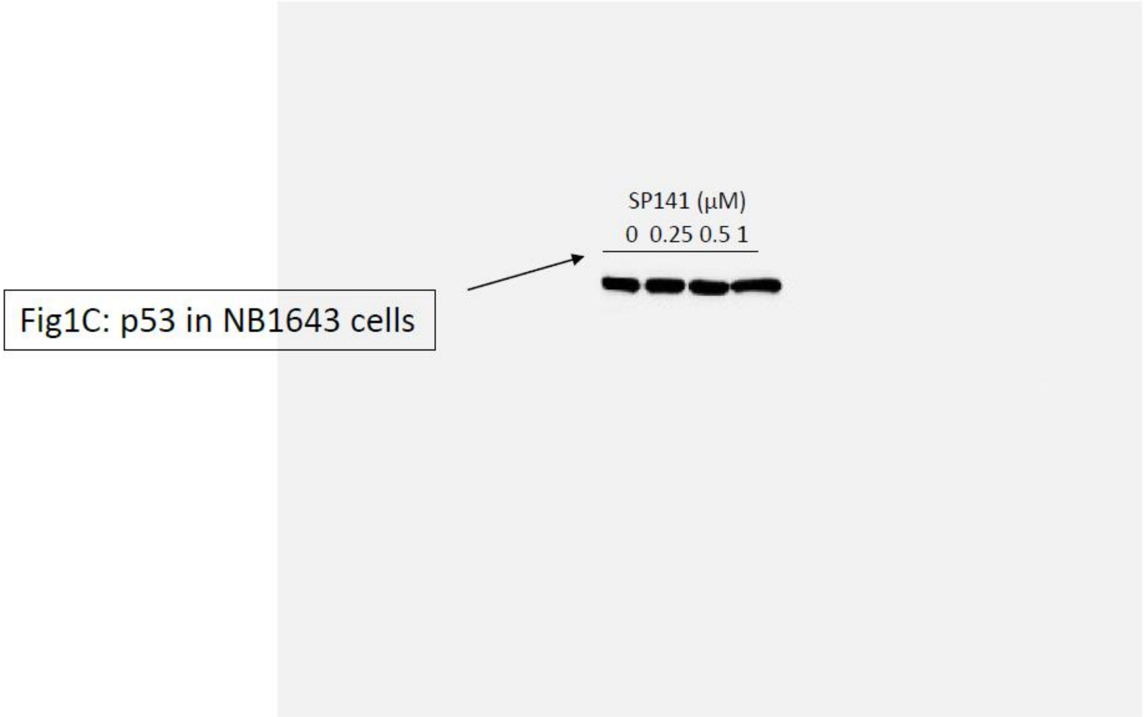

Fig1C: p53 in NB1643 cells

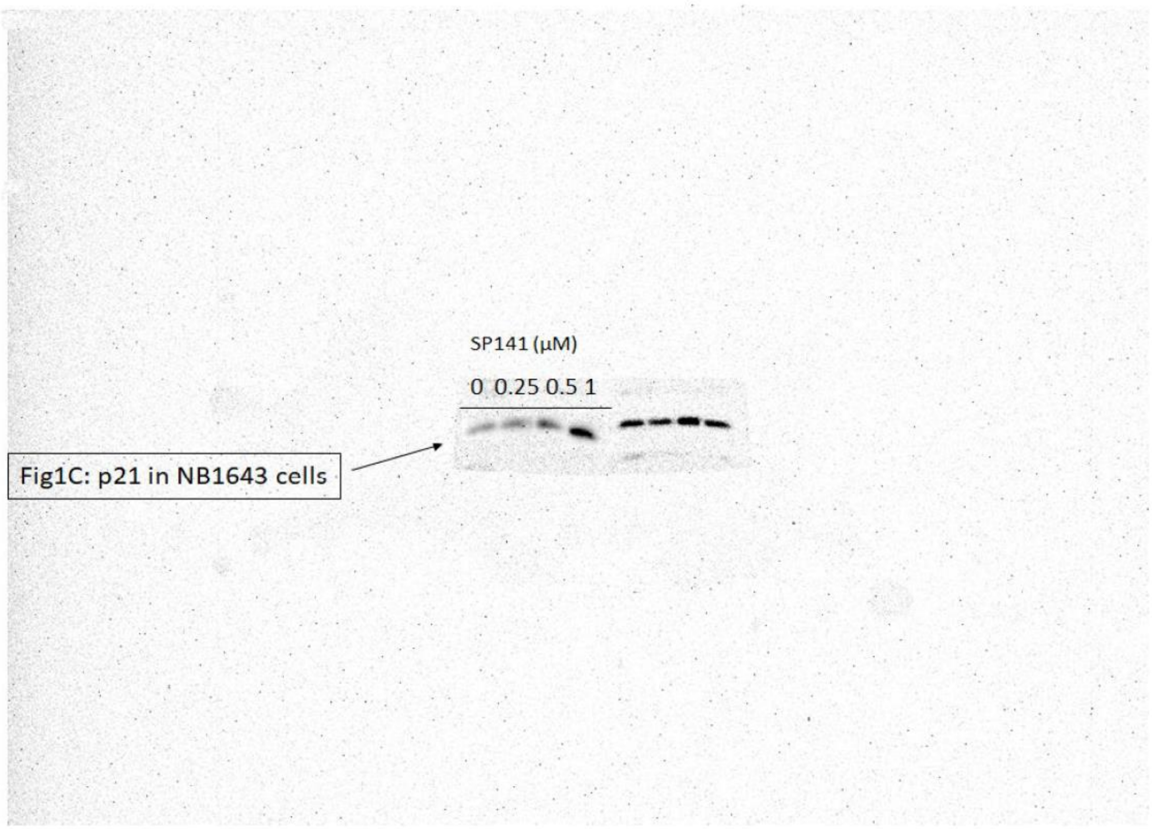
SP141 (µM)
0 0.25 0.5 1
Fig1C: p21 in NB1643 cells

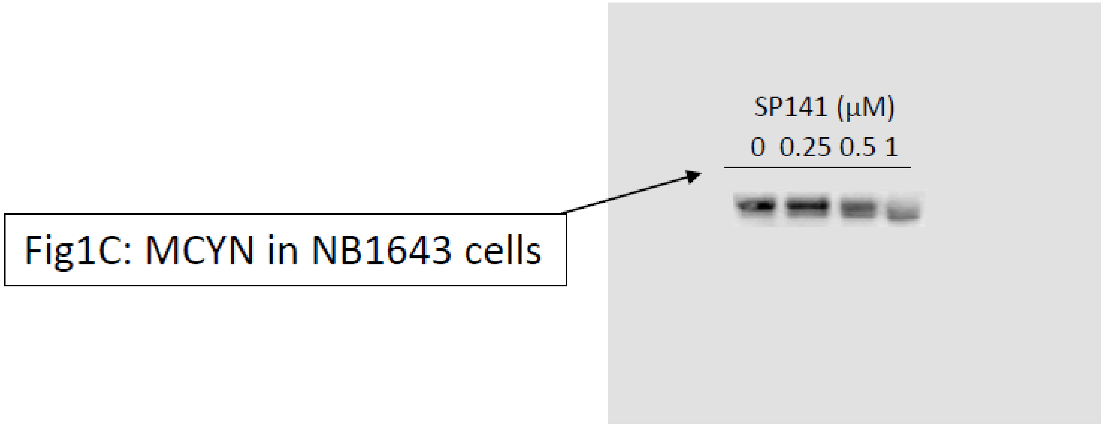
SP141 (µM)
0 0.25 0.5 1
Fig1C: MCYN in NB1643 cells

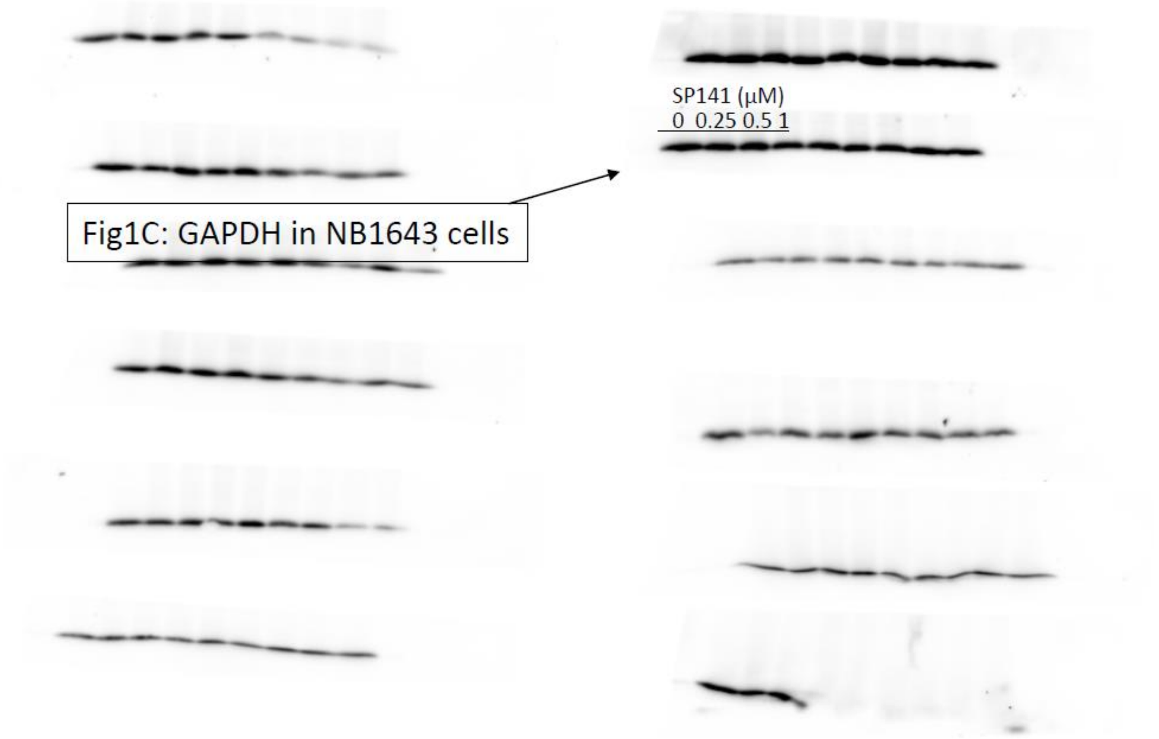
SP141 (μM)
0 0.25 0.5 1
Fig1C: GAPDH in NB1643 cells

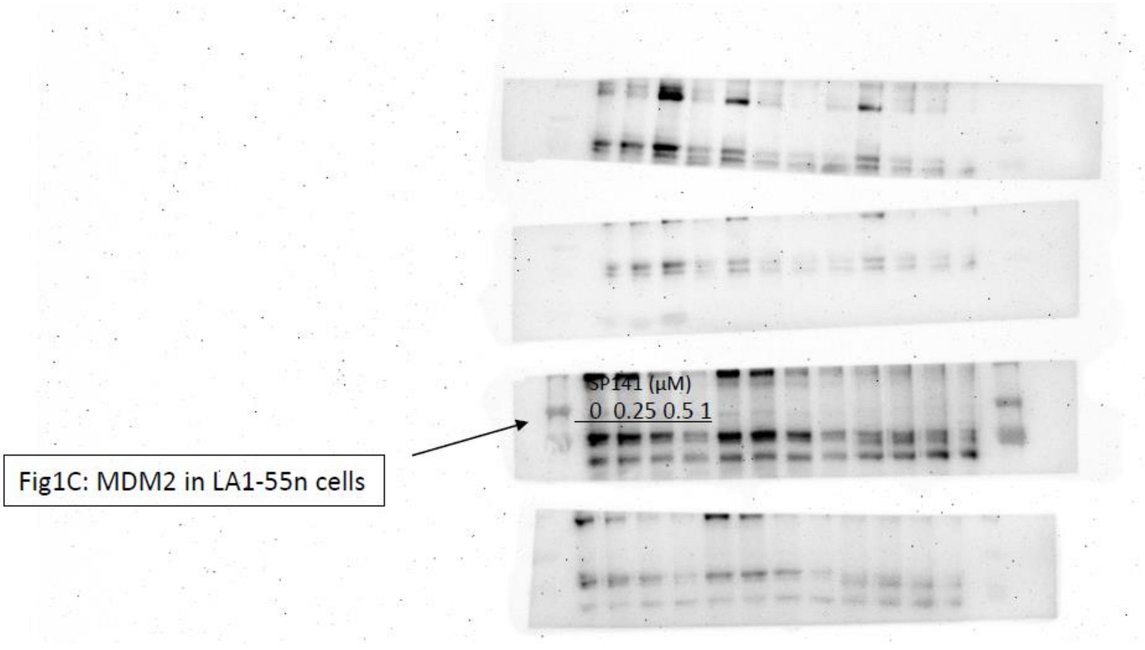
SP141 (μM)
0 0.25 0.5 1
Fig1C: MDM2 in LA1-55n cells

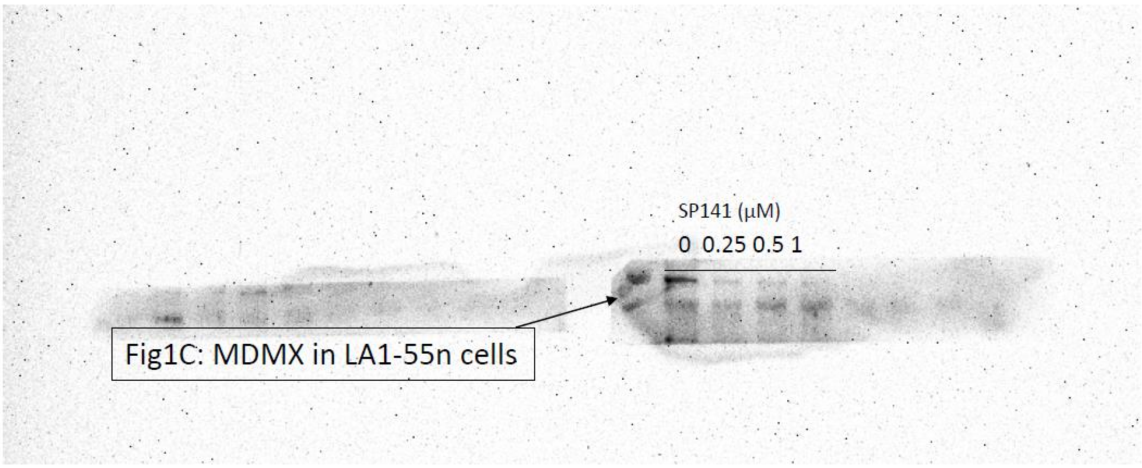
SP141 (μM)
0 0.25 0.5 1
Fig1C: MDMX in LA1-55n cells

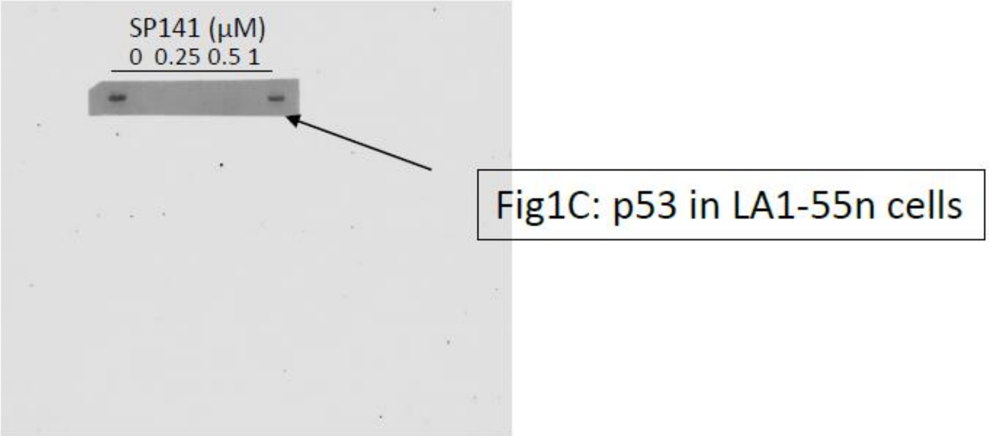
SP141 (μM)
0 0.25 0.5 1
Fig1C: p53 in LA1-55n cells

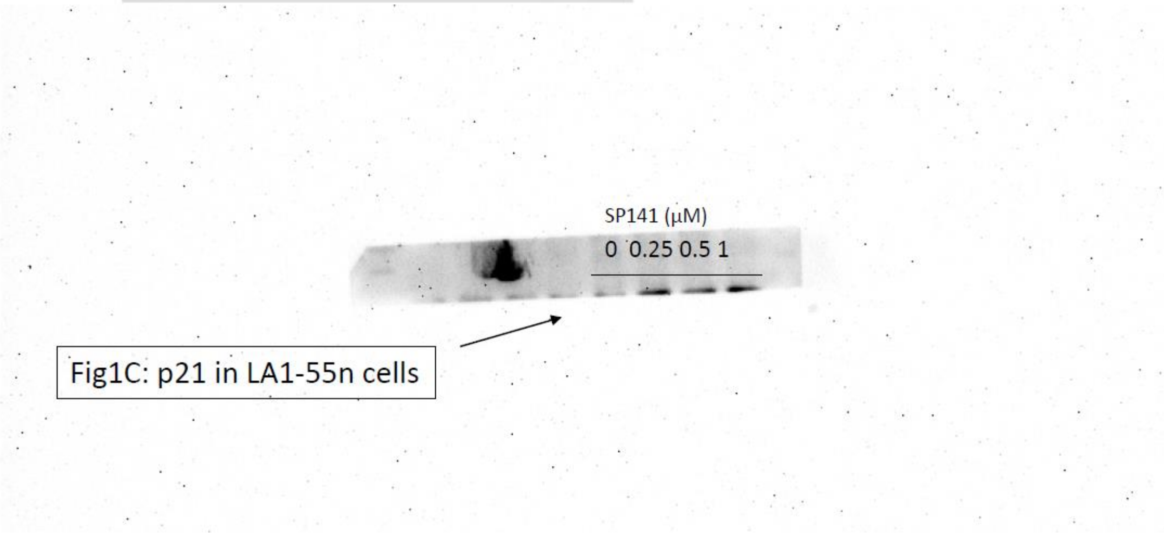
SP141 (μM)
0 0.25 0.5 1
Fig1C: p21 in LA1-55n cells

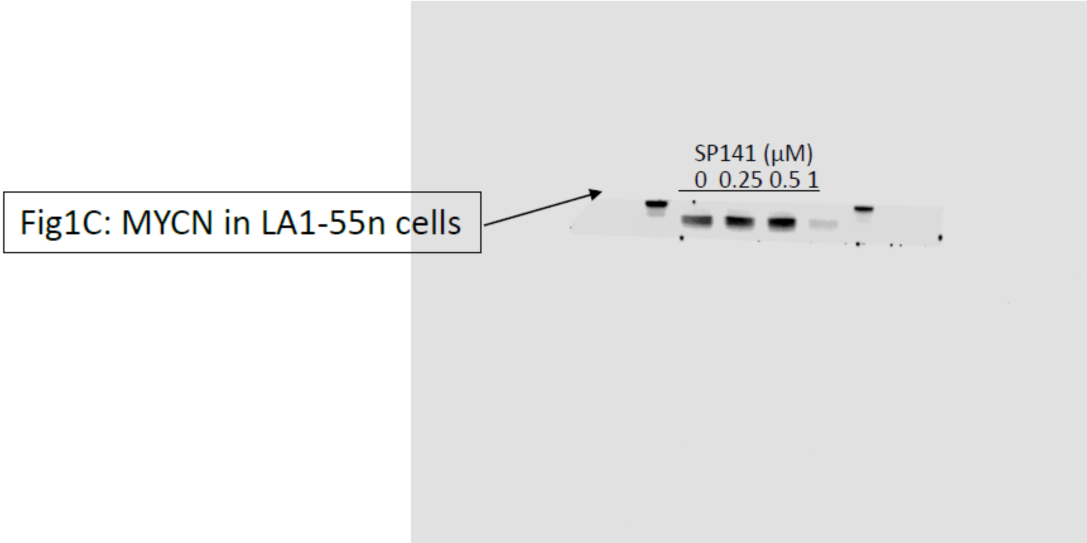
SP141 (μM)
0 0.25 0.5 1
Fig1C: MYCN in LA1-55n cells

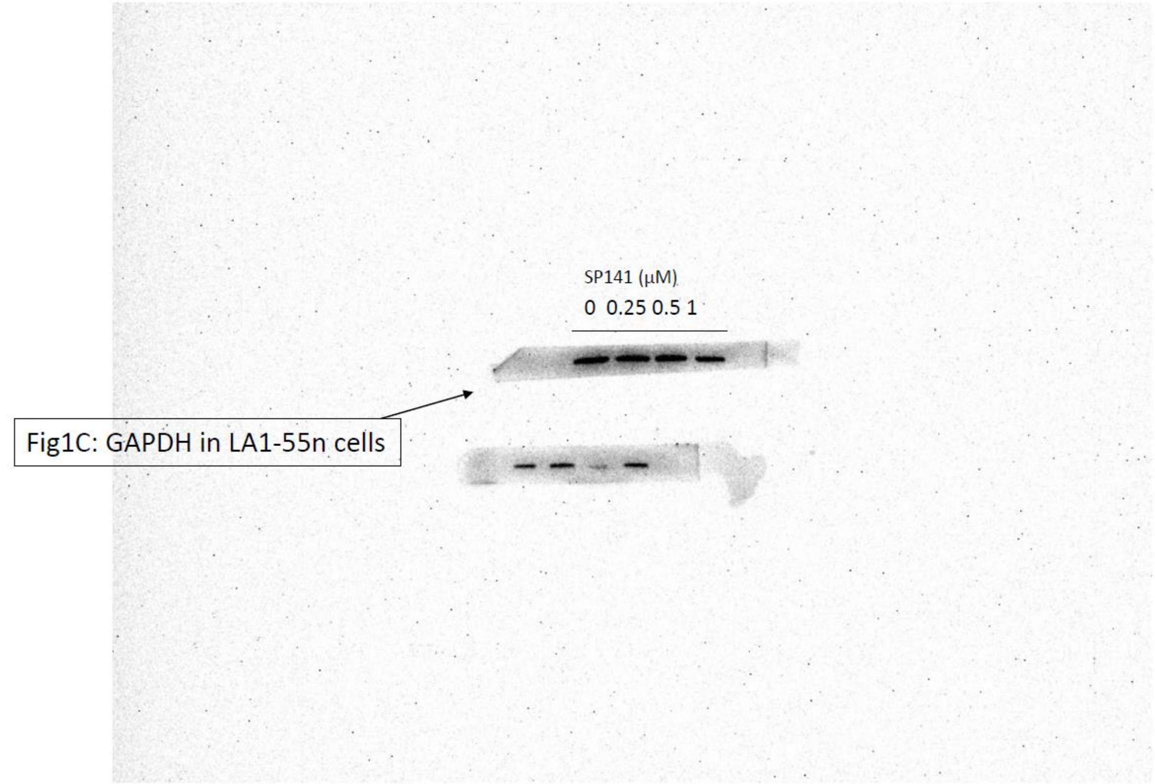
SP141 (μM)
0 0.25 0.5 1
Fig1C: GAPDH in LA1-55n cells

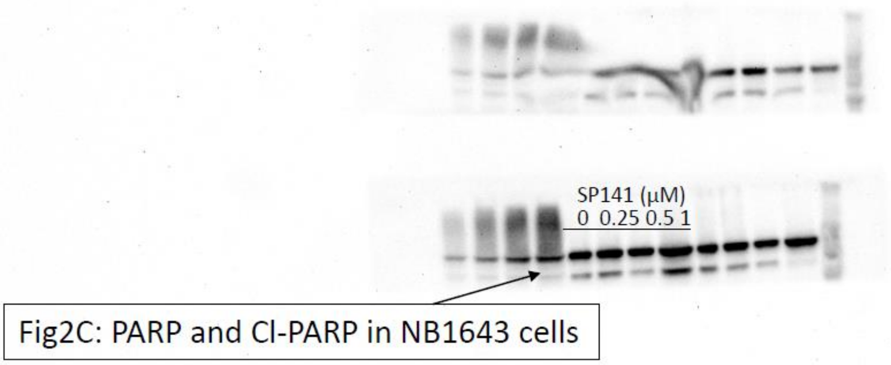
SP141 (μM)
0 0.25 0.5 1
Fig2C: PARP and Cl-PARP in NB1643 cells

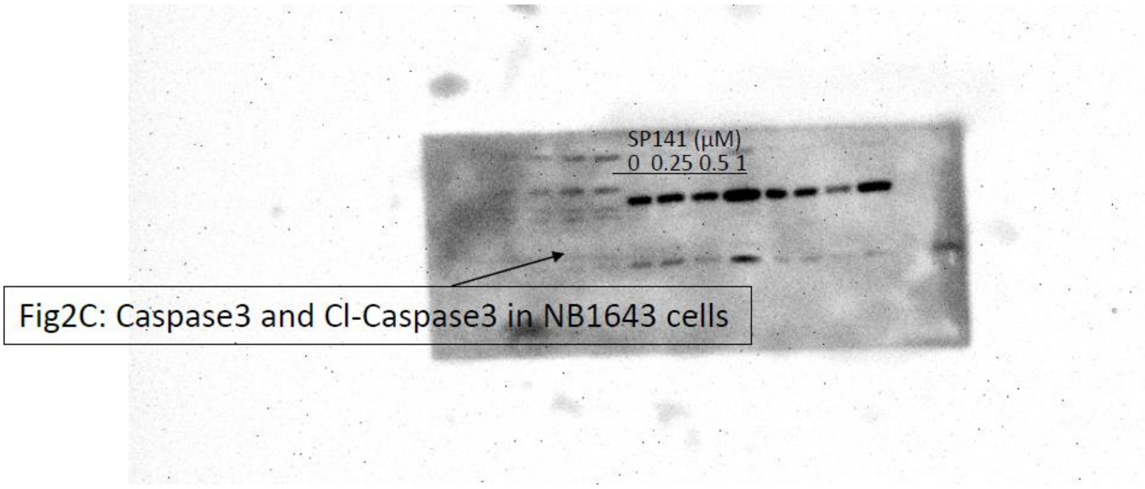
SP141 (μM)
0 0.25 0.5 1
Fig2C: Caspase3 and Cl-Caspase3 in NB1643 cells

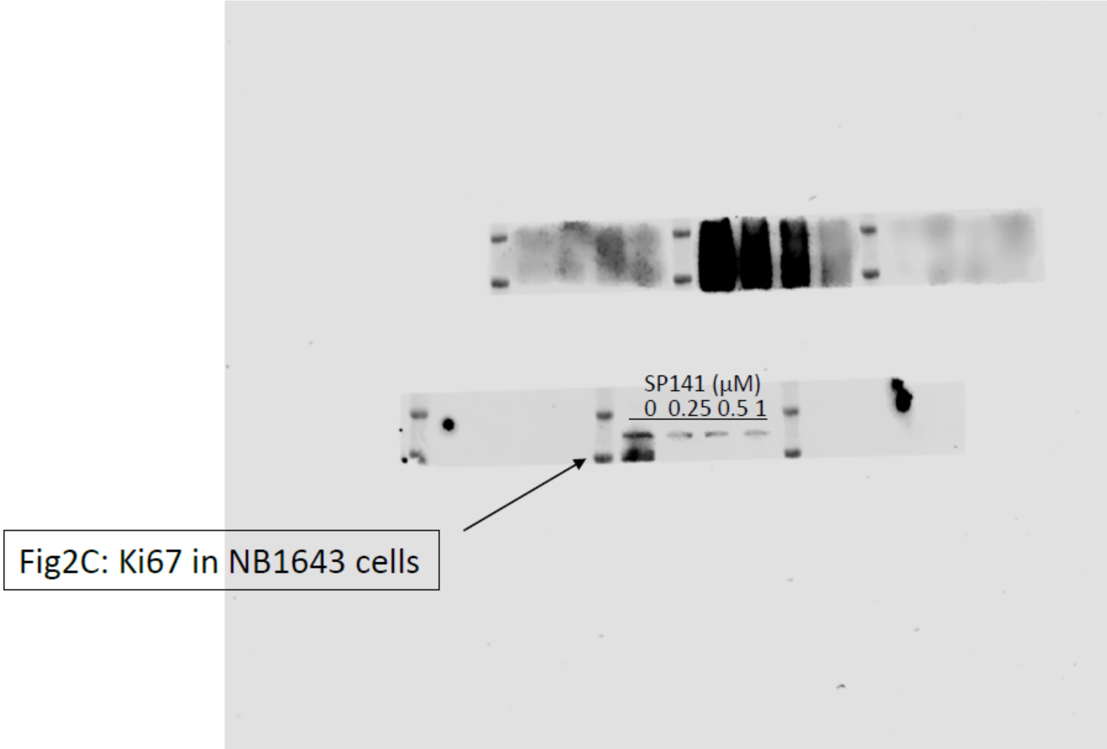
SP141 (μM)
0 0.25 0.5 1
Fig2C: Ki67 in NB1643 cells

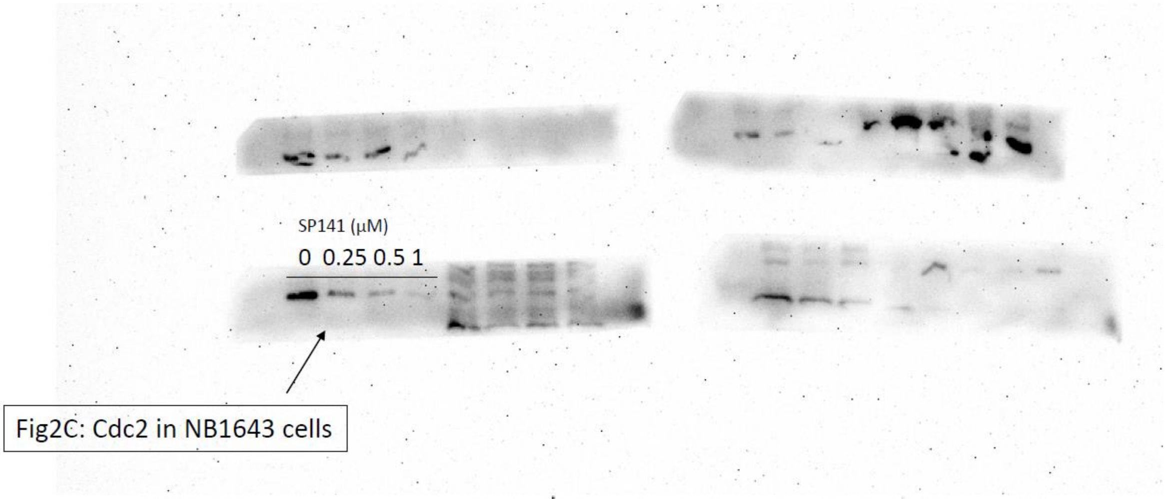
SP141 (μM)
0 0.25 0.5 1
Fig2C: Cdc2 in NB1643 cells

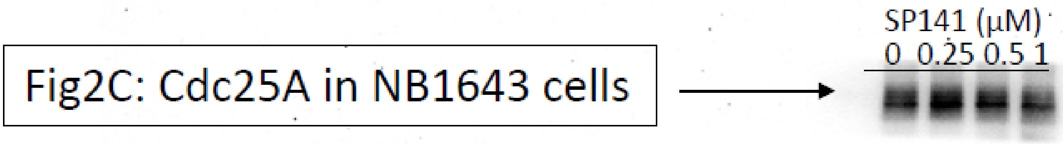
Fig2C: Cdc25A in NB1643 cells
SP141 (μM)
0 0.25 0.5 1

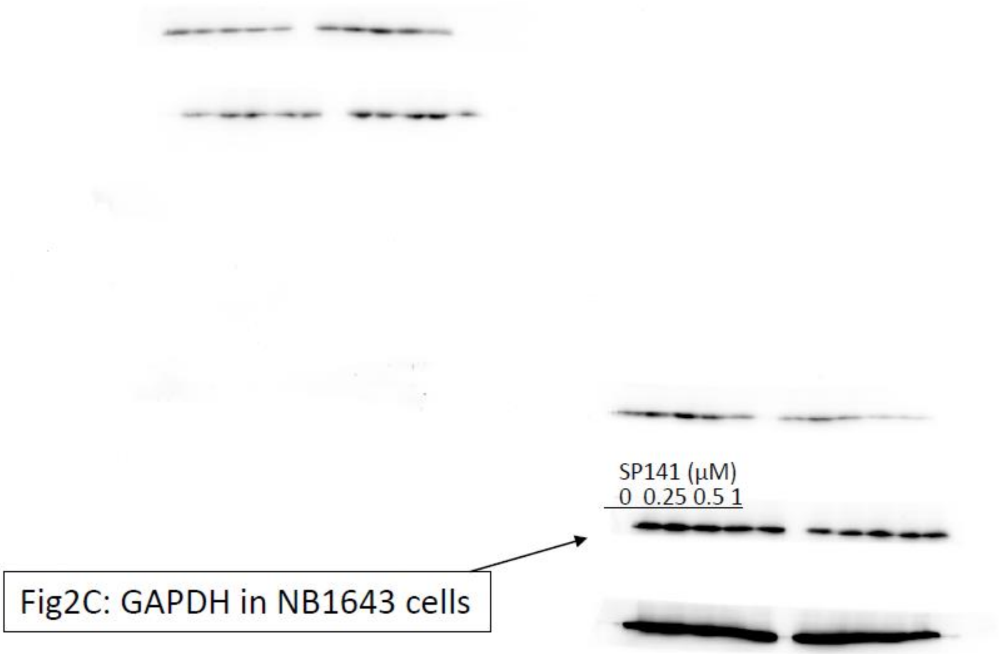
SP141 (μM)
0 0.25 0.5 1
Fig2C: GAPDH in NB1643 cells

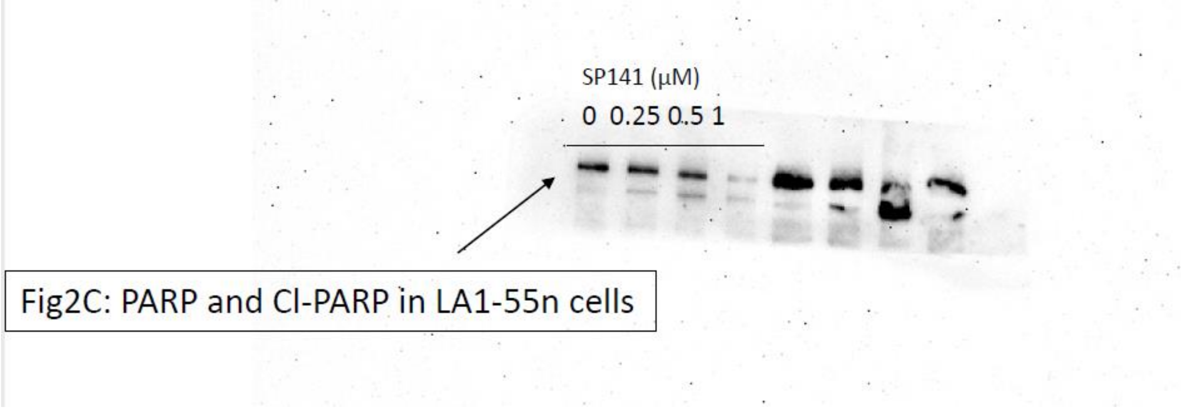
SP141 (μM)
0 0.25 0.5 1
Fig2C: PARP and Cl-PARP in LA1-55n cells

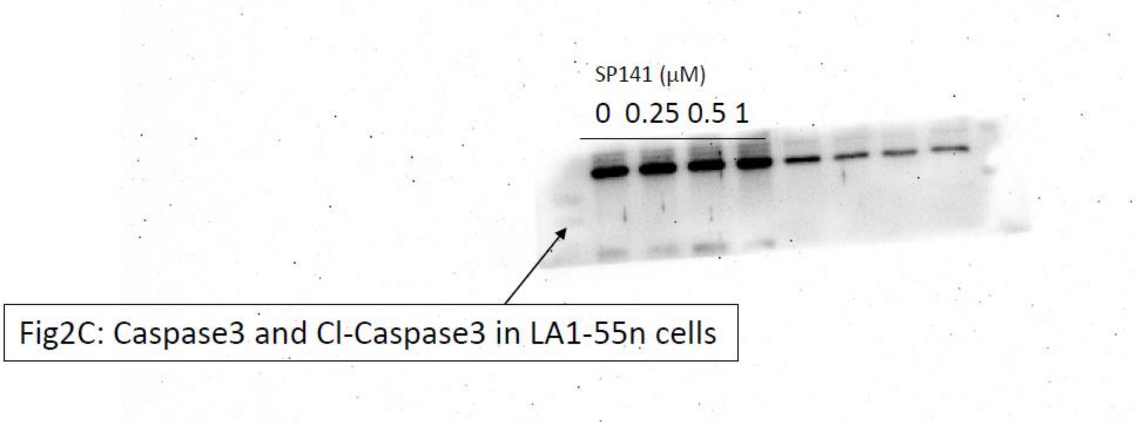
SP141 (μM)
0 0.25 0.5 1
Fig2C: Caspase3 and Cl-Caspase3 in LA1-55n cells

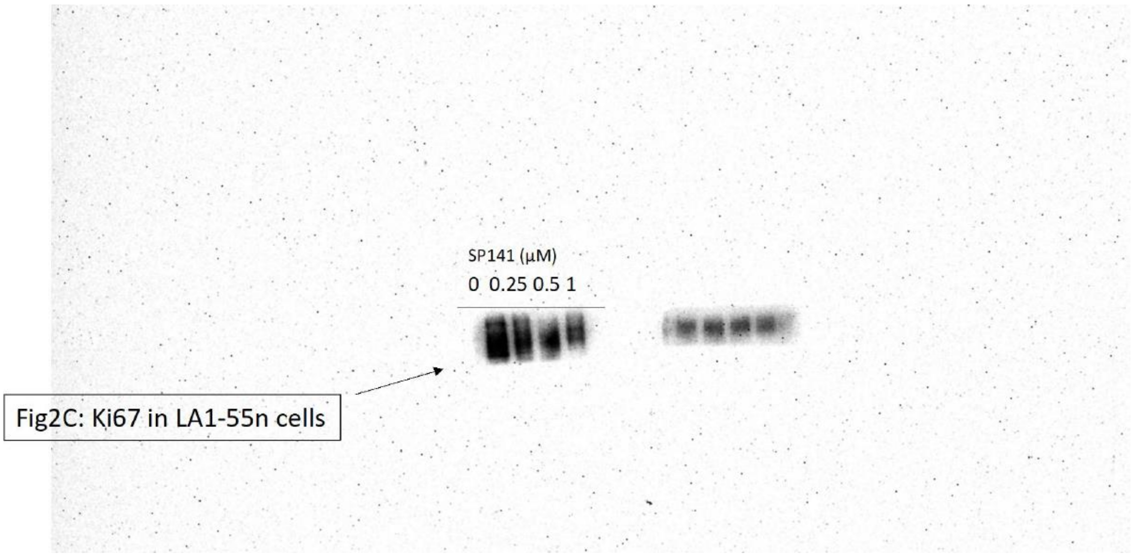
SP141 (μM)
0 0.25 0.5 1
Fig2C: Ki67 in LA1-55n cells

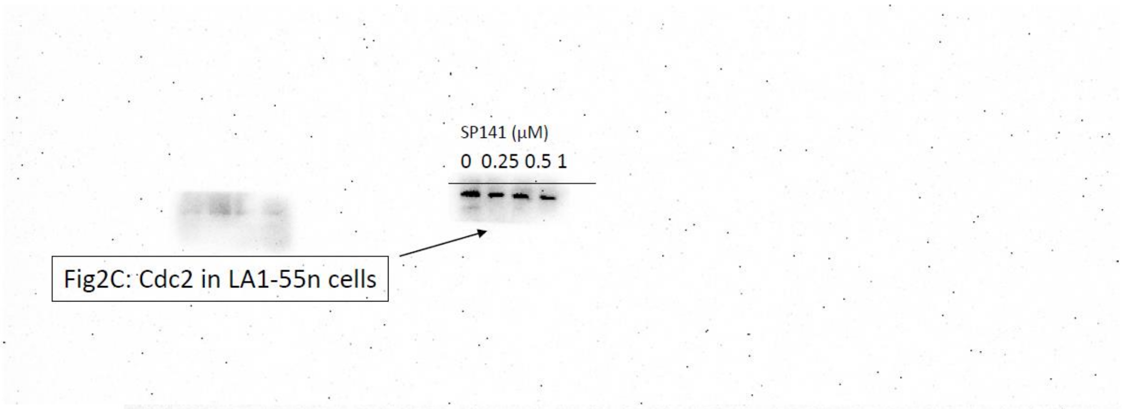
SP141 (μM)
0 0.25 0.5 1
Fig2C: Cdc2 in LA1-55n cells

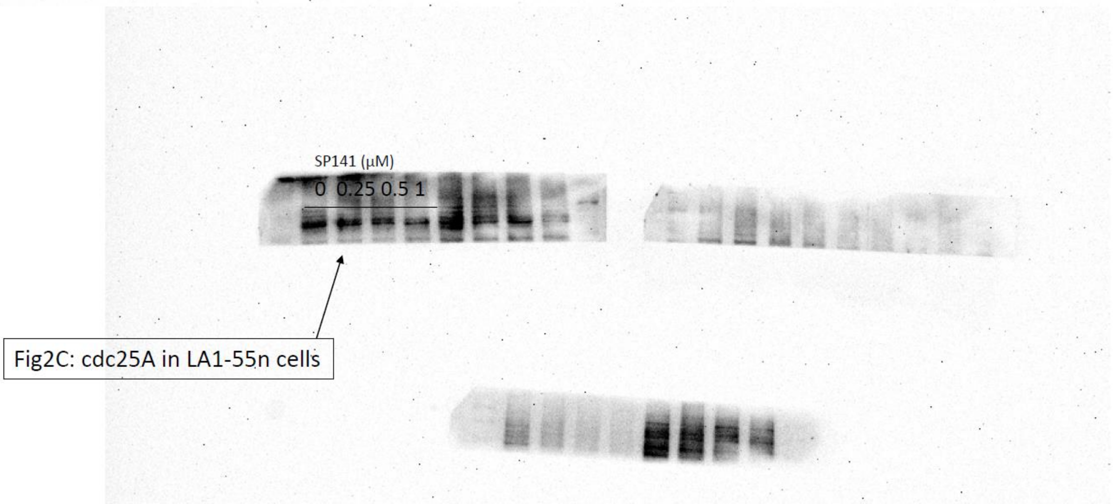
SP141 (μM)
0 0.25 0.5 1
Fig2C: cdc25A in LA1-55n cells

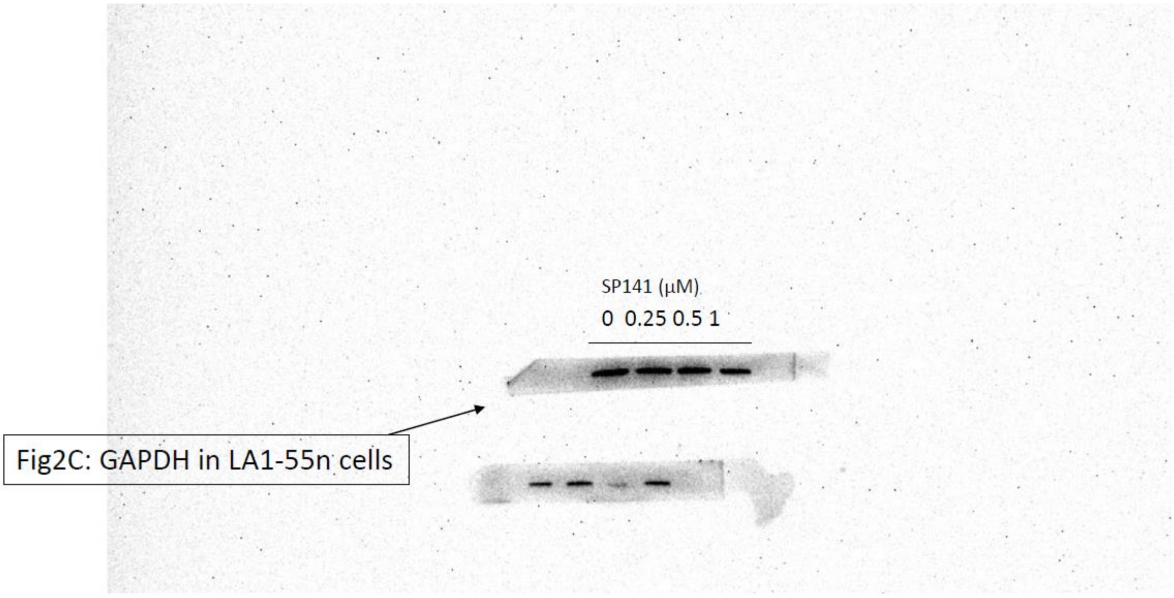
SP141 (μM)
0 0.25 0.5 1
Fig2C: GAPDH in LA1-55n cells

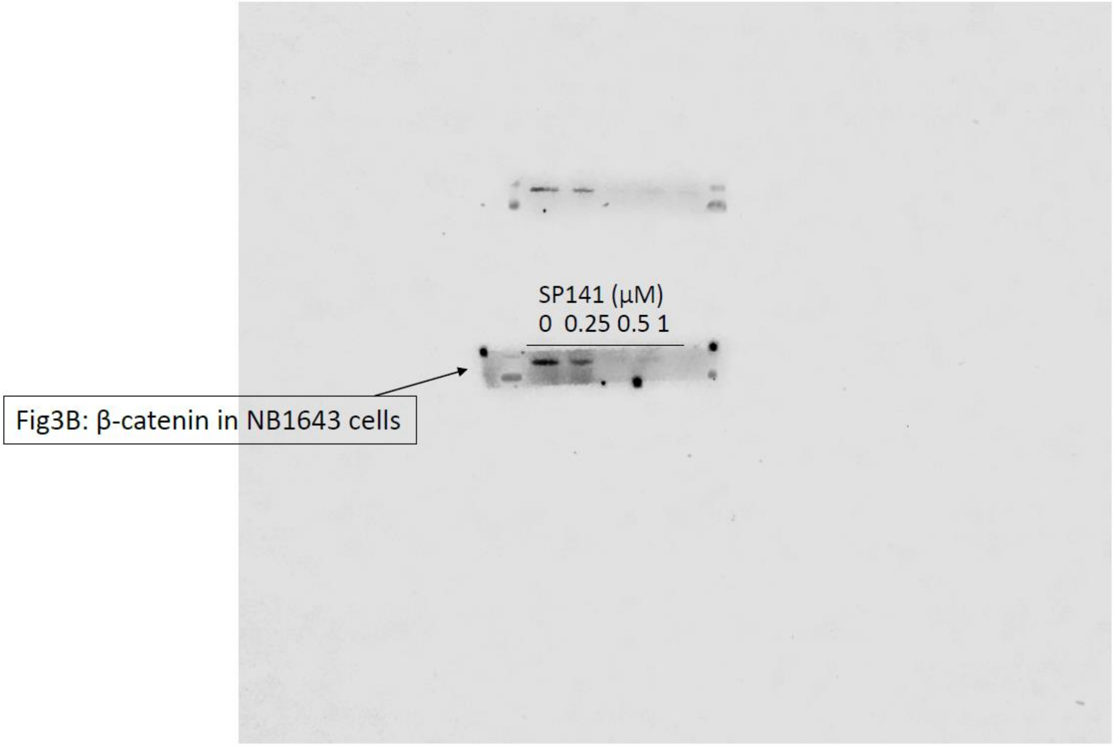
SP141 (μM)
0 0.25 0.5 1
Fig3B: β-catenin in NB1643 cells

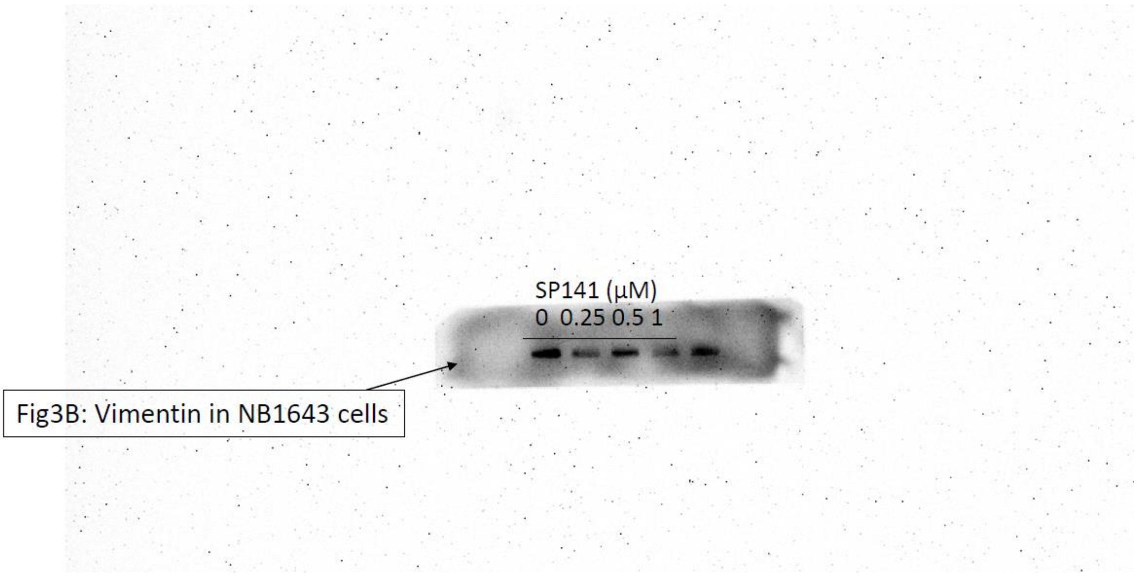
SP141 (μM)
0 0.25 0.5 1
Fig3B: Vimentin in NB1643 cells

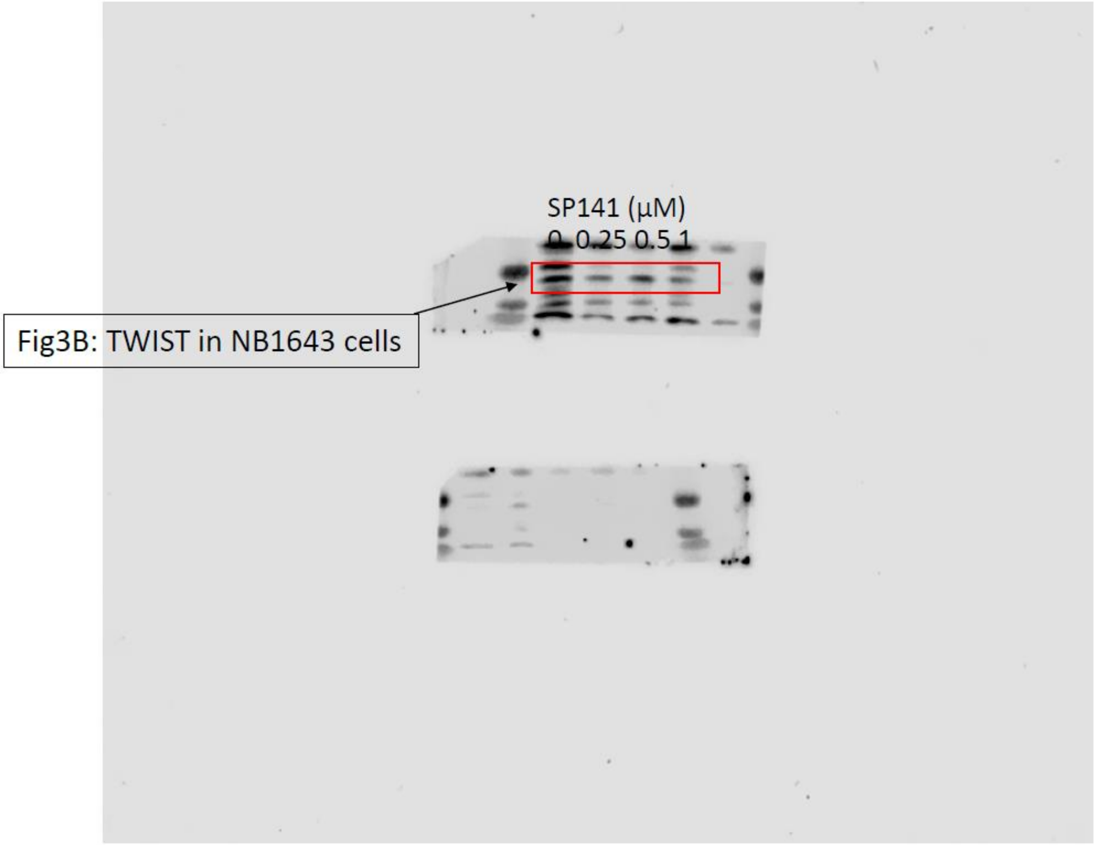
SP141 (μM)
0 0.25 0.5 1
Fig3B: TWIST in NB1643 cells

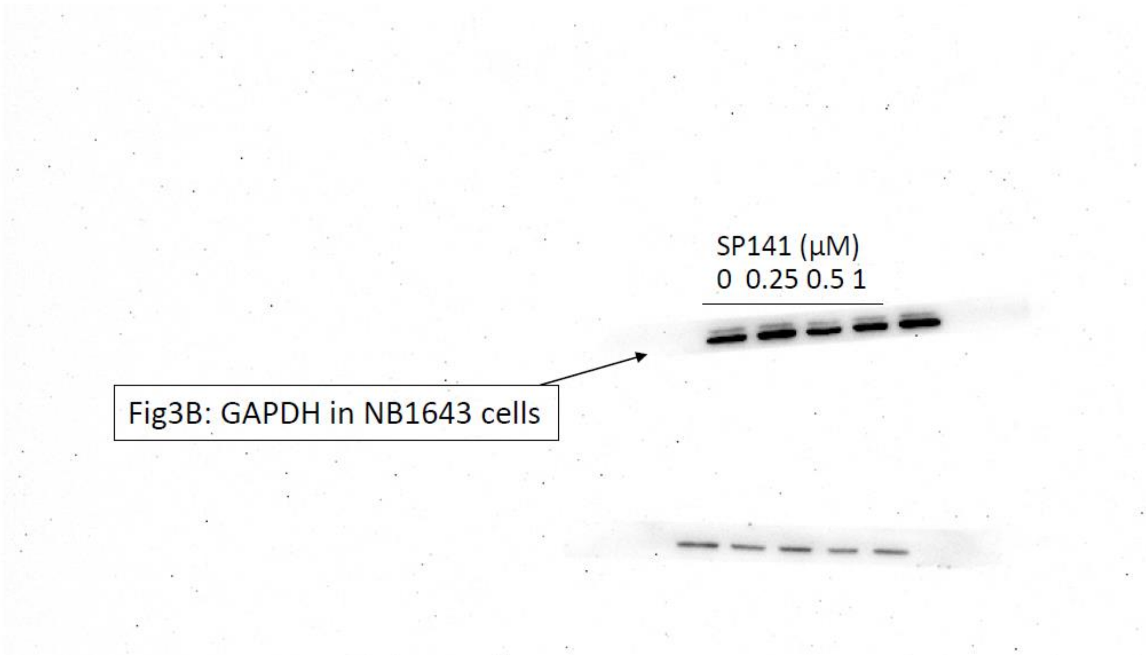
SP141 (µM)
0 0.25 0.5 1
Fig3B: GAPDH in NB1643 cells

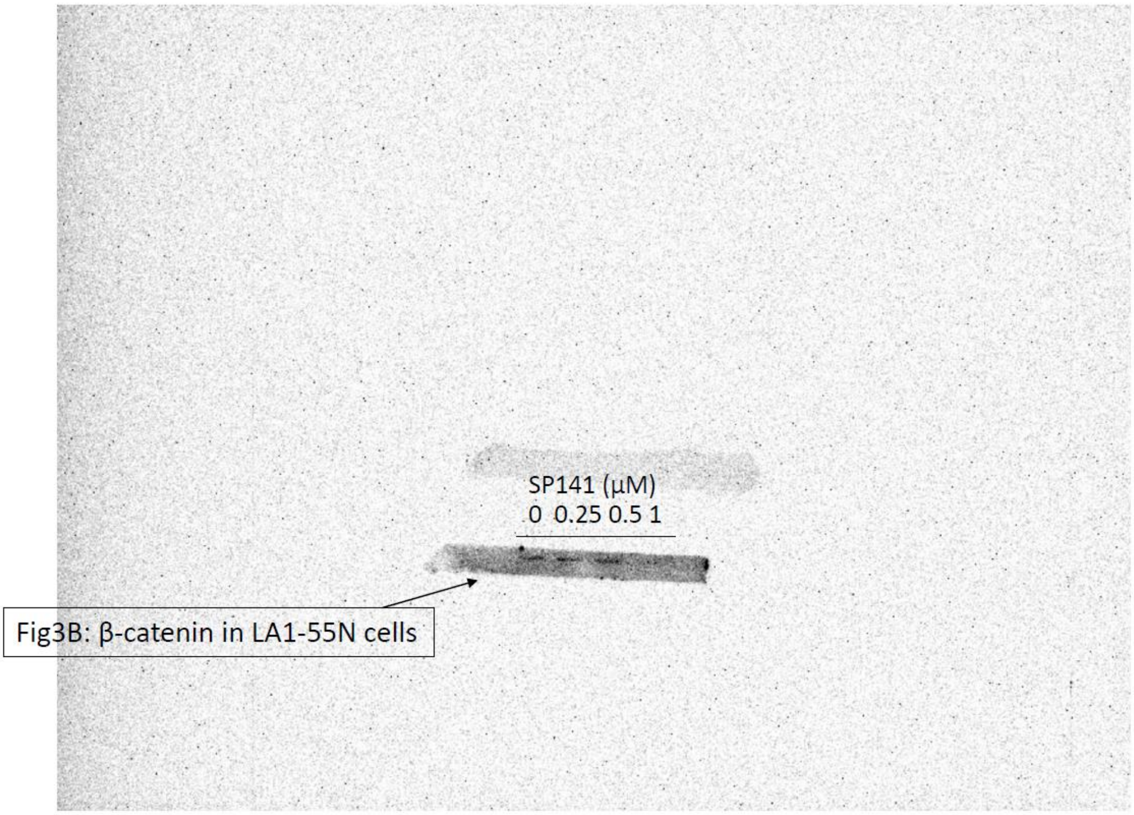
SP141 (µM)
0 0.25 0.5 1
Fig3B: β-catenin in LA1-55N cells

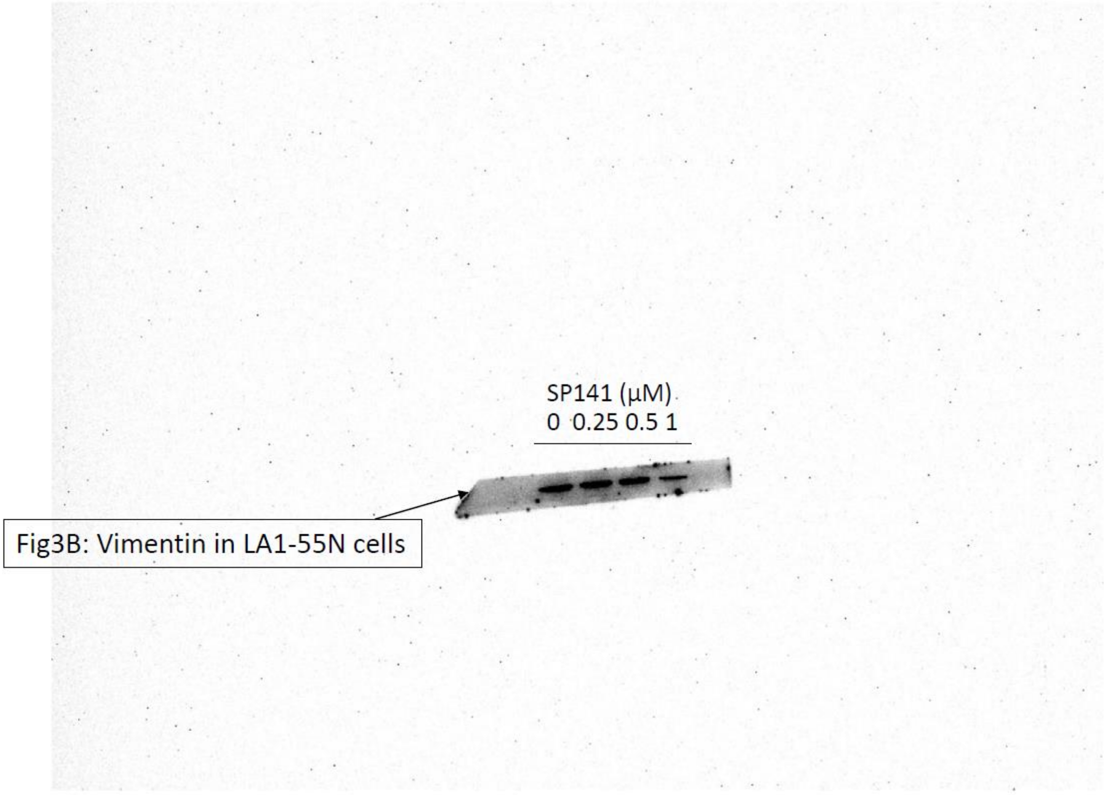
SP141 (μM)
0 0.25 0.5 1
Fig3B: Vimentin in LA1-55N cells

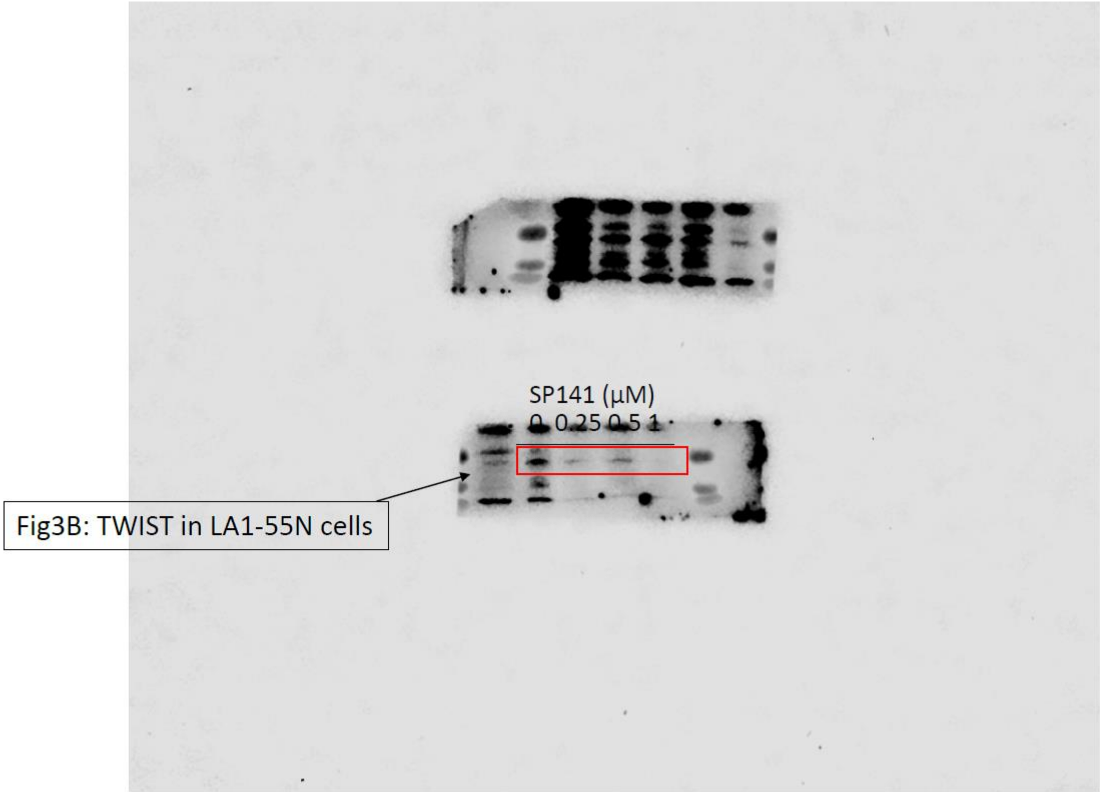
SP141 (μM)
0 0.25 0.5 1
Fig3B: TWIST in LA1-55N cells

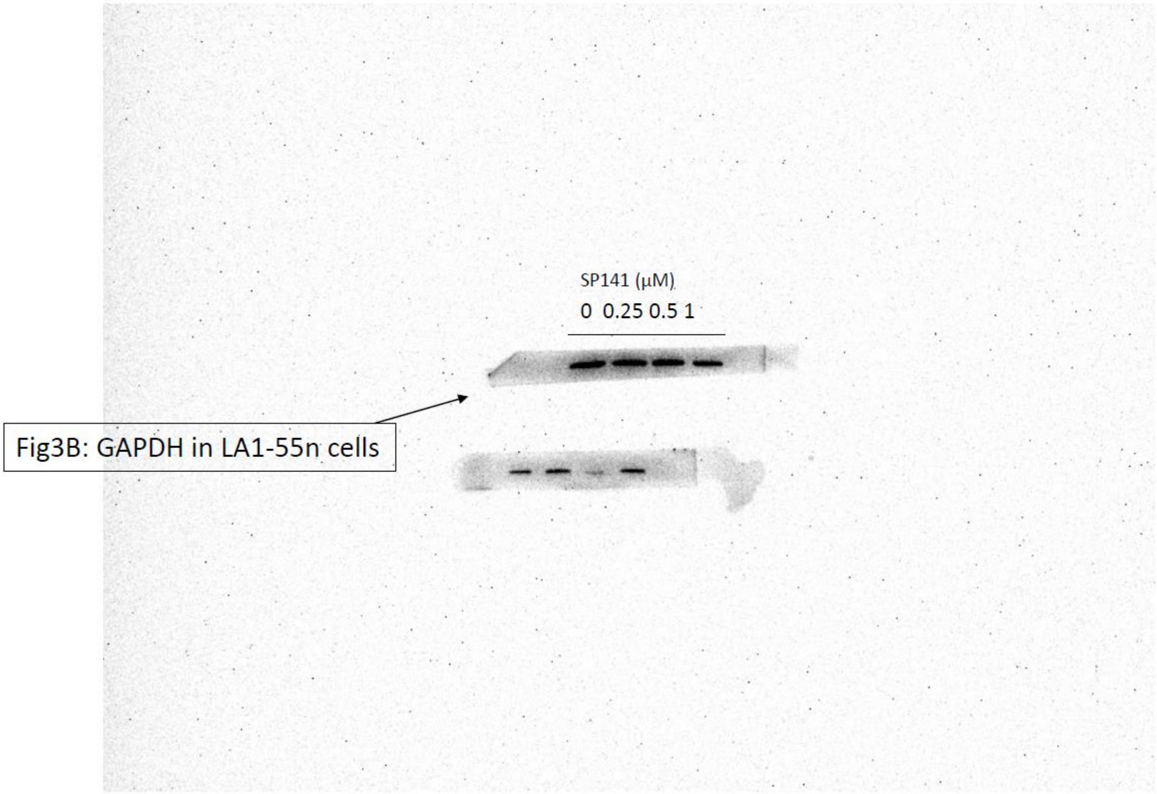

Figure 1. The full western blots.

Publisher's Note: MDPI stays neutral with regard to jurisdictional claims in published maps and institutional affiliations.

© 2020 by the authors. Licensee MDPI, Basel, Switzerland. This article is an open access article distributed under the terms and conditions of the Creative Commons Attribution (CC BY) license (http://creativecommons.org/licenses/by/4.0/).
